# Path Integration and Cognitive Mapping Capacities in Down and Williams Syndromes

**DOI:** 10.3389/fpsyg.2020.571394

**Published:** 2020-12-11

**Authors:** Mathilde Bostelmann, Paolo Ruggeri, Antonella Rita Circelli, Floriana Costanzo, Deny Menghini, Stefano Vicari, Pierre Lavenex, Pamela Banta Lavenex

**Affiliations:** ^1^Institute of Psychology, University of Lausanne, Lausanne, Switzerland; ^2^Department of Neuroscience, Bambino Gesù Children’s Hospital, Rome, Italy; ^3^Faculty of Medicine and Surgery, Catholic University, Rome, Italy; ^4^Faculty of Psychology, Swiss Distance University Institute, Brig, Switzerland

**Keywords:** egocentric, homing behavior, allocentric, cognitive map, spatial memory, navigation, neurodevelopmental disorders

## Abstract

Williams (WS) and Down (DS) syndromes are neurodevelopmental disorders with distinct genetic origins and different spatial memory profiles. In real-world spatial memory tasks, where spatial information derived from all sensory modalities is available, individuals with DS demonstrate low-resolution spatial learning capacities consistent with their mental age, whereas individuals with WS are severely impaired. However, because WS is associated with severe visuo-constructive processing deficits, it is unclear whether their impairment is due to abnormal visual processing or whether it reflects an inability to build a cognitive map. Here, we tested whether blindfolded individuals with WS or DS, and typically developing (TD) children with similar mental ages, could use path integration to perform an egocentric homing task and return to a starting point. We then evaluated whether they could take shortcuts and navigate along never-traveled trajectories between four objects while blindfolded, thus demonstrating the ability to build a cognitive map. In the homing task, 96% of TD children, 84% of participants with DS and 44% of participants with WS were able to use path integration to return to their starting point consistently. In the cognitive mapping task, 64% of TD children and 74% of participants with DS were able to take shortcuts and use never-traveled trajectories, the hallmark of cognitive mapping ability. In contrast, only one of eighteen participants with WS demonstrated the ability to build a cognitive map. These findings are consistent with the view that hippocampus-dependent spatial learning is severely impacted in WS, whereas it is relatively preserved in DS.

## Introduction

Williams syndrome (WS) and Down syndrome (DS, Trisomy 21) are neurodevelopmental disorders of genetic origin, and individuals with these syndromes are generally described as having moderate to severe intellectual disabilities (Ewart et al., 1993; Vicari et al., 2005, 2006; Bittles et al., 2007; Martens et al., 2008). Nevertheless, despite the fact that individuals with these two syndromes have relatively similar IQs [DS: mean 50, range 30-70 (Megarbane et al., 2013); WS: mean 55, range 40-70 (Martens et al., 2008)], these syndromes are characterized by different cognitive profiles. Specific capacities considered to be relatively preserved or a strong point in one syndrome are often more impacted and considered to be a point of weakness in the other (Jarrold et al., 1999; Vicari, 2001; Karmiloff-Smith et al., 2012). The observation of opposite cognitive profiles in individuals with WS and DS has been especially true for spatial capacities. Spatial capacities are a crucial cognitive competence, and impairments in spatial capacities have significant negative impacts on the daily life and autonomy of individuals with intellectual disability. Characterizing the spatial profile of individuals with intellectual disability can thus not only help to identify particular deficits, but also preserved functions that can be targeted to develop syndrome-specific compensatory strategies in order to improve independent navigation (e.g., to go to work, grocery shopping or gather socially), thus increasing self-efficacy, self-confidence and social inclusion.

### Small-Scale Spatial Capacities

Historically, clinical assessments of spatial memory capacities have employed small-scale visuospatial tasks administered using paper-and-pencil [e.g., the Benton line dissection task (Benton et al., 1975)], small apparatuses [e.g., the Corsi block tapping task (Corsi, 1972)] or computers (e.g., Claessen et al., 2015) placed on a desktop directly in front of the individual. Individuals with DS outperform individuals with WS in spatial working memory tasks such as the Corsi block tapping task (Wang and Bellugi, 1993; Jarrold and Baddeley, 1997; Jarrold et al., 1999), or when copying geometric figures (Bellugi et al., 1999). Individuals with DS also outperform individuals with WS on an item-in-location task that requires recalling in which quadrant on a piece of paper an item was previously seen (Vicari et al., 2005).

It has been proposed that global and local attentional capacities (Porter and Coltheart, 2006) or visuo-constructive capacities (the ability to draw or recreate observed visual patterns consistent with their global or local features) are differentially impaired in individuals with WS and DS (Bihrle et al., 1989; Bellugi et al., 1999; Farran et al., 2003); but see D’Souza et al. (2016) for an alternative interpretation. Individuals with DS exhibit relatively better global processing capacities, as compared to their own local processing capacities and the global processing capacities of individuals with WS. In contrast, individuals with WS exhibit relatively better local processing capacities, as compared to their own global processing capacities and the local processing capacities of individuals with DS. However, it is now well-accepted that space is represented in different frames of reference and subserved by distinct yet interconnected brain structures and circuits (White and Mcdonald, 2002; Hartley et al., 2003; Iaria et al., 2003; Burgess, 2006; Banta Lavenex and Lavenex, 2009; Lavenex and Banta Lavenex, 2013; Banta Lavenex et al., 2014; Epstein et al., 2017; Rolls, 2020). Thus, small-scale visuospatial capacities cannot be considered as representative of all spatial capacities for either individuals with typical or atypical development. Indeed, performance on small-scale visuospatial tasks does not necessarily correlate with or predict performance on large-scale spatial tasks in which participants must move around (Quaiser-Pohl et al., 2004; Hegarty et al., 2006; Farran et al., 2010), such as in everyday life.

### Large-Scale Spatial Capacities

Given that the brain represents space in multiple manners, it is not surprising that when navigating in large-scale environments such as in the real world, humans and other animals use a variety of different spatial strategies (White and Mcdonald, 2002; Hartley et al., 2003; Burgess, 2006; Spiers and Maguire, 2007; Bostelmann et al., 2017). Thus, for example, objects and spatial locations can be represented in an egocentric reference frame which codes locations with respect to one’s own body, in a viewpoint-dependent manner (Banta Lavenex and Lavenex, 2009). Egocentric representations enable route learning, or the ability to go from point A to point B via a rather inflexible stimulus-response type of navigation that entails using landmarks and/or a sequence of left or right turns in a fixed manner to reproduce a previously traveled or communicated route (Hartley et al., 2003; Wolbers and Wiener, 2014). Furthermore, landmarks can be used as beacons (i.e., move towards the church, at the church look for the city hall, move towards the city hall, etc.) or associative cues (i.e., at the church turn left, at city hall turn right, etc.) (Waller and Lippa, 2007). In contrast, objects and spatial locations can also be represented in an allocentric reference frame which codes locations in relation to other objects and locations in the environment, in a viewpoint-independent manner (Banta Lavenex and Lavenex, 2009). When using allocentric memory representations, individuals can navigate between objects in their environment in a flexible manner, and are able to take novel, never-before experienced routes or shortcuts to arrive at a desired destination (Tolman, 1948; O’Keefe and Nadel, 1978; Banta Lavenex et al., 2014; Howard et al., 2014; Behrens et al., 2018). As such, the ability to take shortcuts to successfully navigate has come to be regarded as hallmark evidence for the existence of cognitive maps (Epstein et al., 2017; Bostelmann et al., 2020).

Numerous studies have investigated the route learning capacities of individuals with WS or DS in virtual reality environments with local and distal landmarks (Pennington et al., 2003; Farran et al., 2012a, b, 2015, 2016; Courbois et al., 2013; Broadbent et al., 2014, 2015; Davis et al., 2014; Purser et al., 2015; Toffalini et al., 2018). Overall, participants with WS or DS were able to learn the routes, although they sometimes required more trials, or more time, than typically developing (TD) children of the same mental age. Nonetheless, these findings demonstrated that individuals with DS or WS are capable of using an egocentric strategy to solve a route learning task in a virtual environment. In contrast, when required to take a novel most direct route between two locations (i.e., shortcuts), which would provide evidence for flexible cognitive mapping abilities, Courbois et al. (2013) found that only 2 out of 7 participants with DS were able to find the most direct route within ten trials of unguided exploration. It is important to note, however, that since participants were given more than one trial to find the shortcut, it is not even clear whether those who succeeded had built a configural representation of the environment during initial learning or whether they had learned the new path during the several unguided test trials. Similarly, Farran et al. (2015) found that only 10% of participants with DS could successfully find a shortcut between two locations, despite the use of repeated trials which gave participants multiple opportunities to learn the shorter route on their own. For individuals with WS, Farran et al. (2015) found that 35% of their participants with WS were able to find the shorter route during the five unguided trials. Similarly, using a cross-maze design, Broadbent et al. (2014) reported that only 20% of participants with WS were considered to navigate the maze using an allocentric strategy. Overall, whereas participants with WS or DS exhibited some preserved route learning ability in virtual environments, the majority of participants with DS or WS were unable to learn and use the relationships between landmarks encountered in these environments to navigate successfully; in other words, they were unable to build a cognitive map.

### Real-World Spatial Capacities

A number of studies have used a variety of real-world (i.e., not virtual reality) paradigms designed to assess the spatial capacities of individuals with WS or DS. These studies have reported impairments in the ability of individuals with WS to learn a large outdoor route (Farran et al., 2010), to efficiently find rewards in radial arm mazes (Mandolesi et al., 2009; Foti et al., 2015), to use egocentric search strategies efficiently (Smith et al., 2009; Foti et al., 2011), to use geometric cues to reorient in a rectangular room (Lakusta et al., 2010), or to locate an object hidden on an array disconnected from the external environment (Nardini et al., 2008). Surprisingly, these studies revealed that in contrast to what was reported in virtual reality experiments, participants with WS are substantially impaired on real-world spatial tasks. In contrast, fewer studies have investigated the real-world spatial capacities of individuals with DS. In one study that assessed route learning, participants with DS performed as well as TD children, thus exhibiting similar or better performance than in virtual tasks (Meneghetti et al., 2020). In another study that assessed real-world allocentric spatial capacities, although participants with DS were impaired as compared to TD children at locating three reward locations in a paradigm that precluded the use of visual scene matching and egocentric spatial strategies, they were nonetheless capable of orienting in the arena using allocentric cues (Banta Lavenex et al., 2015), thus contradicting findings from virtual reality experiments.

A careful examination of the paradigms previously used to asses spatial capacities reveals that they did not always meet the requirements to unequivocally conclude whether individuals with DS or WS are incapable of creating and using an allocentric spatial representation. First, the failure to take shortcuts in virtual environments cannot be used to infer performance in the real world, where coherent visual, vestibular and proprioceptive information is available. Second, in both virtual and real-world paradigms, care must be taken to eliminate the need to rely on other cognitive processes that may impact task performance (e.g., working memory, linguistic competence, mental rotation or imagined visualization, etc.). Accordingly, none of the above-mentioned studies has provided unequivocal evidence as to whether individuals with WS or DS are capable of integrating the various sources of spatial information normally available in the real world in order to create a cognitive map to navigate in their environment.

In that context, Bostelmann et al. (2017, 2018) assessed the allocentric spatial capacities of individuals with WS and DS in a real-world laboratory paradigm: an open-field arena in which participants had to learn the location of one reward among four visually identical reward locations arranged at the cardinal positions in a 4 m × 4 m enclosure surrounded on three sides by opaque curtains. The reward was always hidden in the same location in the arena, but participants entered and exited the arena by different doors on every single trial. Thus, in order to identify the reward location, participants must employ an allocentric representation of the environment to learn and remember that location in relation to distal objects, and to define their own location relative to the reward location. Individuals with WS were severely impaired on this task: only 17% of the participants with WS tested (mean mental age 5.9 years) could solve the task, whereas 95% of TD children can solve the task from 3 years of age (Ribordy et al., 2013; Ribordy Lambert et al., 2015; Bostelmann et al., 2017). In contrast, 78% of the participants with DS (mean mental age 5.6 years) could solve the task (Bostelmann et al., 2018), thus exhibiting markedly better allocentric spatial capacities than participants with WS. These results provide the clearest evidence to date of the most basic cognitive mapping capacities of individuals with WS and DS, in a real-world laboratory setting enabling the integration of all sources of sensory information normally available when moving freely in the environment. However, because WS is associated with severe visuo-constructive processing deficits, it is unclear whether their impairment is due to abnormal visual processing or whether it reflects an inability to build a cognitive map. What is not known, therefore, is whether individuals with DS or WS are able to rely on self-generated movement information, i.e., in absence of visual information, in order to navigate successfully in a real-world environment.

### Building Spatial Representations Without Vision

Path integration is the ability to use information generated by one’s own body movement, also known as idiothetic cues, to keep track of one’s position in space (Etienne et al., 1996; Mittelstaedt, 1999). Although path integration is often thought of as a mechanism that is used only when visual information is minimized or absent, this is not the case. Path integration is a continuous and automatic process that animals use to determine their position that includes both distance from and direction to their previous position and to other objects and locations in the environment (McNaughton et al., 1996). When visual information is present, path integration is achieved by simultaneously integrating visual, optic flow, vestibular and proprioceptive information. In absence of visual input, angular displacement information (rotation) is provided primarily by vestibular input, and linear displacement information (translation) is provided primarily by proprioceptive input (Etienne et al., 1996; Etienne and Jeffery, 2004; Taube, 2007). Path integration is also often thought of as a mechanism that only allows an individual to return to an original starting point (i.e., home), but this is also not the case. Although path integration can be used to construct an egocentric spatial representation which allows an individual to home, path integration can be used to construct an allocentric spatial representation of the environment (McNaughton et al., 1996; Etienne et al., 1998). However, it is important to note that in absence of external landmarks, path integration is an imprecise process that accrues error with every step (translation) and every turn (rotation). Thus, when using path integration, occasional sensory information from the external environment, such as visual, tactile or olfactory stimuli, must provide a confirmation of the individual’s position, thus eliminating cumulated error and (re)calibrating the path integration system (Klatzky et al., 1990; Fujita et al., 1993; Loomis et al., 1993; McNaughton et al., 1996, 2006; Allen, 2004; Etienne and Jeffery, 2004; Savelli and Knierim, 2019).

Egocentric representations constructed from path integration do not need to incorporate the contextual information from the environment that allows an individual to place themselves in a particular location relative to other objects or locations. As the individual moves along a trajectory, idiothetic information is constantly and automatically encoded. When the individual is ready to return to home, a direct path is calculated, even though the individual may have no knowledge of its location with respect to the surrounding environment other than its starting position. Adults and children from at least 5 years of age are capable of using path integration in absence of visual information to return to a starting point via a direct path, after being led or locomoting along a path that includes one or more turns in what are known as triangle-completion tasks (Corlett et al., 1985; Klatzky et al., 1990; Loomis et al., 1993; Giovannini et al., 2009; Smith et al., 2013; Bostelmann et al., 2020).

**FIGURE 1:**
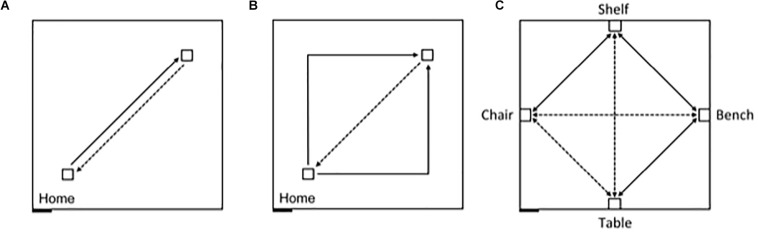
Schematic representation of the experimental design, carried out in an 8 m × 8 m testing room. The black rectangle at the bottom left represents the entry door to the room. Solid lines indicate guided trajectories; dashed lines indicate direct paths that participants were verbally requested to make. **(A)** Egocentric task, straight paths: 7 m straight line guided trajectory, 7 m return path. **(B)** Egocentric task, angled paths: 10 m angular guided trajectory with a right or left turn (5 m + 5 m), and 7 m return path. **(C)** Allocentric task: guided routes (solid) and novel routes (dashed) between four objects, in absence of vision. The paths between the bench and the chair, and between the table and the shelf were 7 m long; the other paths were 5 m long.

In contrast, allocentric representations incorporate the contextual spatial information from the surrounding environment that allows the individual to place themselves in a particular location relative to other objects and locations, as well as to define the position of objects and locations in the environment relative to one another and independently of the observer’s position (McNaughton et al., 1996; Etienne and Jeffery, 2004; Jayakumar et al., 2019; Savelli and Knierim, 2019). Adults have been shown to be capable of using path integration in the absence of vision to create allocentric representations (Passini and Proulx, 1988; Giudice, 2018). And, germane to the present study, Bostelmann et al. (2020) recently showed that TD children between 5 and 9 years of age are also capable of using path integration in absence of visual information to create both egocentric and allocentric representations. In a large 8 m × 8 m empty room, children were first assessed on their ability to return along a 7 m path to a starting location after being led on a straight 7 m outward path, or on a 10 m outward path with a 90° right or left turn in the middle (Figures 1A,B). This experiment thus gave an indication of the children’s ability to integrate their movements to create an egocentric representation, and to orient and walk straight while blindfolded. Ninety-six percent of the TD children tested were able to consistently return to the starting quadrant. In a second experiment, children were blindfolded before entering the same large room that now contained 4 pieces of furniture placed at the midpoint along the walls within the room (Figure 1C). Children were led along direct paths, and asked to navigate independently, between the bench and the shelf, the shelf and the chair, and the bench and the table, respectively. Children were then asked to go directly from the bench to the chair, from the chair to the table, and from the table to the shelf, which required them to take direct, novel shortcuts; and then to retrace the three novel routes in the opposite direction. Sixty-four percent of the 5-9-year-old children tested were capable of using path integration to build a cognitive map enabling them to take shortcuts. Importantly, task performance was not dependent on age, and as many of the younger participants (5-7 years) passed the test as older participants (7-9 years). The authors thus concluded that children from at least 5 years of age are able to use path integration to create a cognitive map of their environment.

### Aim of This Study

The aim of this study was to characterize the ability of individuals with WS and individuals with DS to use path integration in the absence of visual information to build egocentric and allocentric spatial representations enabling them to navigate in their environment. We used the same paradigm previously used with TD children (Bostelmann et al., 2020), and compared the performance of participants with WS or DS to that of TD children with similar mental ages. Performance on the homing task informed us about the ability of participants to walk straight and return to their starting point when blindfolded, as well as to the levels of accuracy and precision that we could expect in the cognitive mapping task.

With vision, most individuals with DS are able to form low-resolution allocentric spatial representations (Bostelmann et al., 2018), whereas a minority of individuals with WS are able to do so (Bostelmann et al., 2017). If cognitive mapping is subserved by the same spatial representational system that underlies performance in the open-field allocentric spatial memory task with vision, then we can predict that a similar proportion of participants with DS will be capable of using path integration to build a cognitive map without vision (about two thirds), whereas very few individuals with WS should be able to do so. However, because WS is associated with severe visuo-constructive processing deficits, it is unclear whether their impairment is due to abnormal visual processing or whether it reflects an inability to build a cognitive map. Thus, precluding access to visual information might reveal the preservation of underlying allocentric spatial capacities in individuals with WS, in which case more individuals with WS may be capable of building a cognitive map using path integration without vision than solving the allocentric spatial memory task with vision.

## Methods

### Participants

We tested eighteen participants with WS (8 females), nineteen participants with DS (9 females) and twenty-eight TD children (15 females) with a chronological age similar to the mental ages of the participants with WS and DS (Table 1). *NB*: The results of the TD children have been published previously (Bostelmann et al., 2020). Participants with WS and DS were recruited in Switzerland (WS: *n* =8; DS: *n* =5) and Italy (WS: *n* =10; DS: *n* =14). According to parents and/or caregivers, none of the participants exhibited signs of age-related dementia. All TD children were recruited in Switzerland. They were reported by their parents to have been typically developing, and were neither born prematurely, nor had any suspected or diagnosed neurological conditions or learning disabilities.

**TABLE 1:** Demographics of participants at the beginning of the study.

		Chronological age (years)				Mental age (years)			
		Mean	*SD*	Min	Max	Mean	*SD*	Min	Max
TD	*n* =28	6.91	1.41	4.83	9.67	-	-	-	-
DS	*n* =19	22.71	6.83	15.33	39.44	5.57	0.75	4.67	6.96
WS	*n* =18	24.28	11.22	9.00	44.92	5.89	0.94	4.42	7.50

Participants were tested on the two tasks on separate days, which were anywhere from one day to several weeks apart. Participants were always assessed on the homing task first and on the cognitive mapping task second. Performance in the cognitive mapping task did not correlate with the interval between the two testing sessions (data not shown). Each experiment lasted approximately 45 min. Testing took place Mondays through Saturdays, between 8:00 A.M. and 6:30 P.M. Human subjects research was approved by the Cantonal Ethics Commission for Human Research (Vaud, Switzerland; protocol no. 60/14), and was in accordance with the code of ethics of the World Medical Association (Declaration of Helsinki) for experiments involving human subjects in research. All participants and/or their parents gave informed written consent.

### Testing Facilities

Testing took place within an 8 m × 8 m room at the University of Lausanne for Swiss participants (Figure 1), and in similar-sized rooms in Nardò for Italian participants with DS and in Fano for Italian participants with WS. During the homing task, the room was devoid of objects. Construction tape was placed on the floor, 1.5 m from each of the walls that constituted the four corners of the room. At the corner closest to the entry door, the tape was arranged to represent a house, which was designated as “home,” i.e., the position to which participants were instructed to always return to on each trial (“go home”). In the other three corners of the room, the tape was arranged in the form of a small “x” surrounded by a square that served as a visual landmark for the experimenter when guiding the blindfolded participants. During the homing task, participants were filmed with a camera on a tripod placed in the far corner of the room opposite the corner containing the home. During the cognitive mapping task, the testing room contained four real-size pieces of furniture. Each object was placed against the center of a wall: a bench (0°), a shelf (90°), a chair (180°), and a table (270°). Participants were filmed with a camera on a tripod placed in the corner of the room between the bench and the table.

Visual information was eliminated with a “sleeping mask” blindfold that was individually adjusted to the participant’s head and face at the start of each trial for the homing task, and before entering the room for the cognitive mapping task. A black scarf was tied around the mask and the participant’s head to ensure that they could not see any light. Two experimenters tested participants. Experimenter 1 (E1) would guide the participant, and Experimenter 2 (E2) recorded the data. E1 walked next to or behind the participant, close enough to provide non-specific verbal encouragement (e.g., “You’re doing great!,” regardless of performance) and to assure their security when they were walking independently (e.g., to prevent them from walking into walls or objects), but far enough so as not to interfere with the participant’s movements. In both tasks, participants with DS or WS were rewarded with coins of small denomination, and children were rewarded with small food rewards (e.g., Smarties^®^, Goldfish^®^ crackers, gummy bears, pieces of breakfast cereal or pretzels, etc.). One reward was given for each completed trial and was not based on performance. Children’s parents were queried with respect to alimentary allergies, and children were asked whether there were any treats that they did not like.

### Specific Testing Procedures

#### Homing Task

Participants were tested on their ability to return to a starting point after being led along a predetermined route. Before beginning, participants were told that they would be guided along some paths while blindfolded, and that it was their job to try to return to the starting point as precisely as possible at the end of each guided route. Participants were instructed that once they thought that they were at the home location they were to stop walking and remain stationary. Each participant performed 4 sets of 5 trials without vision. Half of the trials consisted of a linear route of 7 m (Figure 1A), and the other half of the trials consisted of a 10 m route with a 90° left or right turn in the middle (Figure 1B). The trials were given in the following order: Straight path session 1: 5 × 7 m linear route, guided by the left arm; Two-legged path session 1: 5 × 10 m route with a 90° right turn at the halfway point, guided by the left arm; Straight path session 2: 5 × 7 m linear route, guided by the right arm; Two-legged path session 2: 5 × 10 m route with a 90° left turn at the halfway point, guided by the right arm. At the end of each guided route, and while still facing in the direction of the outbound travel, E1 released the participant’s arm and instructed them to “go home” (to the starting point). Although participants had been instructed to stop walking once they estimated that they had arrived at home, if participants were approaching a wall and did not show signs of stopping, E1 gently placed a hand on the participant to stop them. Once participants were stationary, they could take off the blindfold, look where they were positioned in the room, and then return to the starting position in order to prepare for the next trial. To ensure that all participants understood the task, prior to the beginning of each session they experienced a practice trial during which they were led through the guided part of the path without the blindfold, their arm released at the end of the guided path, and then asked to “go home.” A trial was terminated when a participant stopped alone or when the experimenter stopped the participant just before a wall.

#### Cognitive Mapping Task

Participants were tested on their ability to take novel paths (shortcuts) to navigate to previously visited locations marked by large stable objects. In this task, four objects were placed in the 8 m × 8 m room (Figure 1C). Prior to entering the room, participants were told that they were going to be blindfolded, and that they would then explore our laboratory’s living room. Participants were never told the goal of the experiment, or that they would have to remember the positions of the objects in the room or navigate to those objects using novel routes. Although all participants were familiar with the room from having participated in the homing task, they were blindfolded prior to entering the room for the cognitive mapping task, and thus never saw the objects or their positions in the room. Once blindfolded, participants were led into the room and were guided to the bench where they were asked to sit down. Importantly, although the bench was located on the far right-hand wall relative to the entry door, some participants may not have had explicit knowledge of its absolute location in the room; it could just as easily be perceived as being on the far wall opposite the door.

##### Learning Phase

Participants were taught the routes between (1) the bench and the shelf, (2) the shelf and the chair, and (3) the bench and the table, always in the same order for each participant (Supplementary Material 1). Accordingly, at the beginning of each trial, participants were positioned so that they were either sitting straight on the bench or chair, or so that their back was touching the shelf or the table, and their feet pointing straight forward. For each route to be learned, participants were guided by the arm round-trip between the two objects twice by E1, then asked to make the round-trip alone one time, then guided through two more round-trips, and finally asked to make two more round-trips alone (for a total of four guided and three non-guided round-trips per route). Each time participants reached an object by themselves or when guided by E1, E1 named the object and participants were asked to touch the object or sit on it, for the chair and the bench.

In non-guided trials, if a participant came within 30 cm of the target object, E1 would gently take their arm and guide them into contact with the object, so that the participants would not startle or injure themselves colliding with the object, thus terminating the trial. If the participant was in the correct quadrant of the room (within an arc of 45° extending from the starting object and centered around the target object; tape markings on the floor outlined this zone), but not within 30 cm of the target object, the participant was allowed to continue walking until they came within 30 cm of a wall, at which point E1 gently stopped the participant and guided them to the target object. If a participant began walking in the wrong direction and after traveling 4 m was not in the correct quadrant, E1 would gently stop them and guide them back to the starting object, and then begin escorting the participant through the next two guided trials.

##### Testing Phase

Participants ended the learning phase sitting on the bench, and immediately began the testing phase from there. Participants were asked to walk alone and directly to objects, which would require them to take novel paths or shortcuts to these objects. First, E1 asked participants to walk directly from the bench to the chair (“now, go alone directly to the chair”). Once sitting in the chair, they were asked to walk directly to the table. Once their back was to the table, they were asked to walk directly to the shelf. Then, participants were asked to perform the three reverse routes: from the shelf to the table (“now, go alone directly to the table”), from the table to the chair, and from the chair to the bench. In the testing phase, each trial and data collection terminated when a participant either: (1) came within 30 cm of the target, at which point E1 gently guided the participant to the object, or (2) came within 30 cm of a wall, at which point E1 gently stopped the participant and guided them to the target object.

### Data Collection

Participants’ movements and trajectories were recorded with the Noldus TrackLab system (Wageningen, Netherlands). Participants wore a vest on which a radio frequency-emitting Ultra-Wide Band tag was affixed to each shoulder. The system collected the X and Y coordinates of each tag at a frequency of 4.75 Hz. The smoothed averaged X and Y coordinates of the two tags were computed to plot the location of the participant’s head on a 2D representation of the room. Each trajectory was then transferred to the ImageJ program (NIH, United States), and retraced to measure the distance and angle information for the different parts of each individual trajectory.

We used several measures to quantify participants’ performance on each trial: (1) The initial heading, defined as the angular difference between the ideal path and the participant’s path one meter after starting their journey. (2) The final heading, defined as the angular difference between the ideal path and the participant’s path after the participant either stopped alone (homing task), reached the target object (cognitive mapping task), or was stopped by E1 (homing and cognitive mapping tasks). (3) The distance to target, defined as the shortest distance between the participant’s final position and home, or the target object. For the homing task, the five trials of each session without vision were averaged to obtain one single value for each of these measures (1-3) for each participant. For the cognitive mapping task, the six novel paths were analyzed separately.

We also provided an overall measure of task performance: (4) “Pass” or “Fail.” For the homing task, we estimated whether participants passed or failed by determining whether their average end location was within the quadrant of the room that included the outbound journey’s starting point, as defined by the two perpendicular bisectors of the room’s walls. In the cognitive mapping task, we estimated whether participants passed or failed each of the three novel paths and the three reverse paths. To be considered as passing, the end point of the participant’s trajectory had to be within the same quadrant as the target object, as defined by the two diagonals bisecting the room. This defined the area of the room in which participants were closer to the target object than any other object. We did not use a more restrictive criterion (within an arbitrary distance to the target), because even young adults do not exhibit perfect performance and do not always come within contact-distance of the object at the end of their trajectory (Bostelmann et al., 2020).

### Data Analysis

For angular measures of direction, we used circular statistics computed in Excel following the formulas described by Zar (1999). To quantify the variability of individual participants within each session of the homing task, we computed the length of the mean vector (i.e., of the average angle) for each participant ($l_{j}=\sqrt{X^{2}+Y^{2}}$, where $X=\sum_{i=1}^{n}\cos\alpha_{i}/n$ and $Y=\sum_{i=1}^{n}\sin\alpha_{i}/n$). Since the average value of l across all participants was 0.97 ± 0.04, and to give the same weight to the results of each individual participant, we considered this value to be 1 for the computation of second-order group analyses.

We performed one-sample tests for the mean angle to determine whether each group’s average initial or final heading followed a mean heading that deviated from the perfect angle. We considered a 99% confidence interval to define a significant departure from the ideal direction. Group comparisons were performed with the Watson-Williams test for angular measures. We reported the groups’ angular deviation, $s=\frac{180}{\pi }\,\sqrt{2\,\left(1-r\right)}$, in order to provide a measure of inter-individual variability within each group (Zar, 1999; pp. 602-605). We further compared the groups’ angular deviation based on the absolute differences of the rectangular coordinates between individual average angles and an ideal heading angle of zero: $a\,b\,s_{j}=\sqrt{{\left(\sin\alpha_{j}-0\right)}^{2}+{\left(\cos\alpha_{j}-1\right)}^{2}}$.

Statistical analyses were performed with IBM SPSS Statistics for Macintosh, version 25.0 (IBM Corp. Armonk, NY, United States). We used General Linear Model analyses with groups as a between-subject factor and test sessions or trials as repeated measures to analyze the distance to target, and the variability of initial and final headings. We used independent samples t-tests to compare performance between two groups. We used Chi^2^ tests to compare the number of participants in different groups that passed the tasks. The level of statistical significance was set at *p* < 0.05 for all analyses.

## Results

### Homing Task: Straight Outbound Paths

#### “Pass” or “Fail”

**FIGURE 2:**
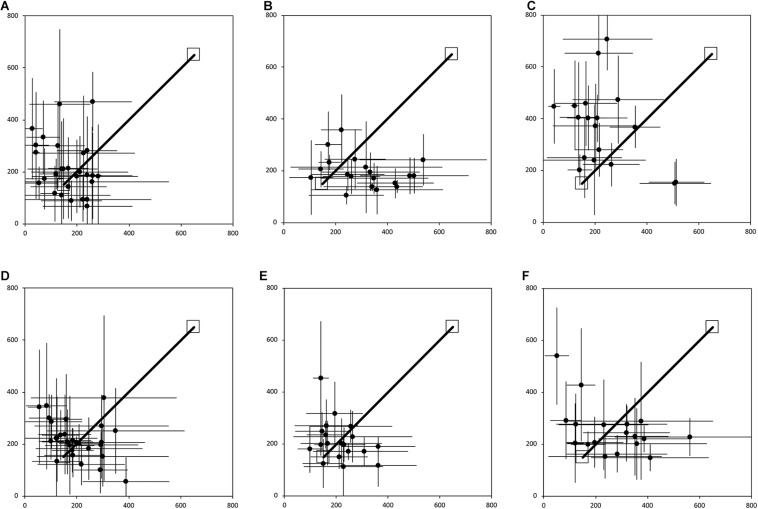
Average end location of individual participants’ return paths, following a straight 7 m outward path in the egocentric homing task. The horizontal and vertical error bars represent the standard deviation for each individual across one session. The solid line indicates the outward straight path. The top right square indicates the starting point of the return path. The bottom left square indicates “home.” In Session 1, participants were guided by the left arm, and in Session 2 they were guided by the right arm. **(A)** TD, Session 1. **(B)** DS, Session 1. **(C)** WS, Session 1. **(D)** TD, Session 2. **(E)** DS, Session 2. **(F)** WS, Session 2. Room size: 800 cm × 800 cm.

Figure 2 shows the average end locations of TD children, participants with DS, and participants with WS who were asked to return to their starting point after being led blindfolded on a straight 7 m path, thus ideally requiring a 180° turn and a 7 m straight walk to return to the starting point. For Session 1 (Figures 2A-C), 93% of TD children, 74% of participants with DS and 39% of participants with WS had an average end location in the quadrant of the room where the home was located (Tables 2-4). Thus, in Session 1, fewer participants with WS were considered to have passed the homing task than both TD children (*X*^2^ = 15.740, *p* < 0.001) and participants with DS (*X*^2^ = 4.560, *p* = 0.033). The difference between participants with DS and TD children failed to reach the predefined level of statistical significance (*X*^2^ = 3.283, *p* = 0.070). For Session 2 (Figures 2D-F), 100% of TD children, 95% of participants with DS and 78% of participants with WS had an average end location in the quadrant of the room where the home was located (Tables 2-4). Thus, in Session 2, fewer participants with WS were considered to have passed the homing task than TD children (*X*^2^ = 6.815, *p* = 0.009), whereas there were no differences between the number of participants with DS and the number of participants with WS (*X*^2^ = 2.275, *p* = 0.131) or TD children (*X*^2^ = 1.506, *p* = 0.219).

**TABLE 2:** Individual performance of TD children in the homing task.

Participant	Gender	Age (years)		Criterion	S-1	S-2	A-1	A-2
TD39	F	4.83		Pass	Pass	Pass	Pass	Fail
TD160	M	4.83		Pass	Pass	Pass	Pass	Pass
TD167	M	4.83		Pass	Fail	Pass	Pass	Pass
TD187	F	5.00		Pass	Pass	Pass	Pass	Pass
TD50	M	5.50		Pass	Pass	Pass	Pass	Fail
TD53	F	5.50		Pass	Pass	Pass	Fail	Pass
TD186	F	5.67		Pass	Pass	Pass	Pass	Pass
TD27	M	5.83		Pass	Pass	Pass	Pass	Pass
TD36	M	5.92		Fail	Fail	Pass	Pass	Fail
TD34	F	6.67		Pass	Pass	Pass	Pass	Pass
TD35	M	6.67		Pass	Pass	Pass	Pass	Pass
TD48	F	6.67		Pass	Pass	Pass	Pass	Pass
TD141	F	6.67		Pass	Pass	Pass	Pass	Pass
TD42	M	6.92		Pass	Pass	Pass	Pass	Pass
TD49	F	7.08		Pass	Pass	Pass	Pass	Pass
TD143	F	7.08		Pass	Pass	Pass	Pass	Pass
TD31	F	7.17		Pass	Pass	Pass	Pass	Pass
TD25	M	7.25		Pass	Pass	Pass	Pass	Pass
TD191	M	7.50		Pass	Pass	Pass	Pass	Pass
TD26	F	7.92		Pass	Pass	Pass	Pass	Pass
TD29	F	7.92		Pass	Pass	Pass	Pass	Pass
TD52	M	8.08		Pass	Pass	Pass	Fail	Pass
TD37	M	8.25		Pass	Pass	Pass	Pass	Pass
TD138	F	8.42		Pass	Pass	Pass	Pass	Pass
TD28	F	8.58		Pass	Pass	Pass	Pass	Pass
TD30	F	8.92		Pass	Pass	Pass	Pass	Pass
TD142	M	9.17		Pass	Pass	Pass	Pass	Pass
TD43	M	9.67		Pass	Pass	Pass	Pass	Pass
			Pass	27	26	28	26	25
			Fail	1	2	0	2	3
			% Pass	96%	93%	100%	93%	89%

*S-1, First session with straight outbound paths; S-2, Second session with straight outbound paths; A-1, First session with angled outbound paths; A-2, Second session with angled outbound paths; Pass, average final location within the target quadrant; Fail, average final location not within the target quadrant. Criterion, Passing on 3 of 4 sessions.*

**TABLE 3:** Individual performance of participants with DS in the homing task.

Participant	Gender	M. A.	C. A.		Criterion	S-1	S-2	A-1	A-2
DS19	M	4.67	16.42		Fail	Fail	Pass	Pass	Fail
DS6	M	4.75	21.17		Pass	Fail	Pass	Pass	Pass
DS8	F	4.75	21.92		Fail	Fail	Pass	Pass	Fail
DS1	M	4.83	28.58		Pass	Pass	Pass	Pass	Fail
DS5	F	5.00	15.33		Pass	Fail	Pass	Pass	Pass
DS13	M	5.00	19.67		Pass	Pass	Pass	Pass	Fail
DS12	M	5.08	22.25		Pass	Pass	Pass	Pass	Pass
DS30	M	5.25	36.17		Pass	Pass	Pass	Pass	Pass
DS24	M	5.29	15.50		Pass	Pass	Pass	Pass	Pass
DS7	M	5.33	18.25		Pass	Pass	Pass	Pass	Pass
DS15	F	5.33	17.75		Pass	Pass	Pass	Pass	Pass
DS23	M	5.58	23.92		Pass	Pass	Pass	Fail	Pass
DS25	F	5.88	20.75		Pass	Pass	Pass	Fail	Pass
DS27	F	6.00	21.17		Pass	Pass	Pass	Fail	Pass
DS26	F	6.21	39.42		Fail	Fail	Fail	Pass	Pass
DS2	F	6.67	18.17		Pass	Pass	Pass	Pass	Pass
DS4	F	6.67	27.42		Pass	Pass	Pass	Pass	Pass
DS22	M	6.67	30.50		Pass	Pass	Pass	Pass	Pass
DS21	F	6.96	17.17		Pass	Pass	Pass	Fail	Pass
				Pass	16	14	18	15	15
				Fail	3	5	1	4	4
				% Pass	84%	74%	95%	74%	74%

*S-1, First session with straight outbound paths; S-2, Second session with straight outbound paths; A-1, First session with angled outbound paths; A-2, Second session with angled outbound paths; Pass, average final location within the target quadrant; Fail, average final location not within the target quadrant. Criterion, Passing on 3 of 4 sessions.*

**TABLE 4:** Individual performance of participants with WS in the homing task.

Participant	Gender	M. A.	C. A.		Criterion	S-1	S-2	A-1	A-2
WS13	F	4.42	9.00		Pass	Fail	Pass	Pass	Pass
WS2	M	4.75	23.92		Pass	Pass	Pass	Pass	Pass
WS15	M	4.75	26.92		Pass	Fail	Pass	Pass	Pass
WS22	F	5.00	14.30		Fail	Pass	Pass	Fail	Fail
WS3	M	5.33	12.83		Pass	Pass	Pass	Pass	Pass
WS5	F	5.33	19.00		Fail	Fail	Pass	Pass	Fail
WS18	F	5.54	23.42		Pass	Fail	Pass	Pass	Pass
WS10	F	5.92	35.08		Fail	Fail	Fail	Pass	Fail
WS17	M	6.00	11.83		Fail	Fail	Pass	Fail	Pass
WS7	M	6.21	26.67		Fail	Fail	Pass	Fail	Fail
WS8	M	6.21	16.17		Fail	Fail	Pass	Fail	Pass
WS4	M	6.67	44.92		Fail	Fail	Pass	Fail	Fail
WS9	F	7.00	15.25		Pass	Pass	Pass	Pass	Fail
WS1	F	7.08	27.17		Fail	Pass	Fail	Pass	Fail
WS12	M	7.08	21.83		Pass	Pass	Pass	Pass	Pass
WS16	M	7.50	42.75		Pass	Pass	Fail	Pass	Pass
WS20	M	-	21.08		Fail	Fail	Fail	Fail	Pass
WS21	F	-	44.83		Fail	Fail	Pass	Fail	Pass
				Pass	8	7	14	11	11
				Fail	10	11	4	7	7
				% Pass	44%	39%	78%	61%	61%

*S-1, First session with straight outbound paths; S-2, Second session with straight outbound paths; A-1, First session with angled outbound paths; A-2, Second session with angled outbound paths; Pass, average final location within the target quadrant; Fail, average final location not within the target quadrant. Criterion: Passing on 3 of 4 sessions.*

#### Average Distance From Home

They were differences between the groups of TD children, participants with DS and participants with WS (*F*_(2,62__)_ = 7.933, *p* = 0.001) and between sessions (*F*_(__2,62__)_ = 18.411, *p* < 0.001), but no interaction between groups and sessions (*F*_(__2,62__)_ = 2.088, *p* = 0.133; Table 5). In both sessions, the distance between the end location and home was greater for participants with WS than for participants with DS and TD children. In contrast, the distance between the end location and home did not differ between participants with DS and TD children. The distance from home was shorter in Session 2 than in Session 1 when all groups were considered together (*t*_(64)_ = 3.912, *p* < 0.001), for the group of participants with WS (*t*_(__17)_ = 2.387, *p* = 0.029) and the group of participants with DS (*t*_(__18)_ = 3.063, *p* = 0.007), but not for the group of TD children (*t*_(__27)_ = 1.359, *p* = 0.185). It is important to note, also, that they were differences between groups in the number of times participants were stopped by the experimenter because they were approaching a wall (*F*_(2,62)_ = 4.427, *p* = 0.016; out of 10 trials; TD: 3.96 ± 2.62 (average ± standard deviation), DS: 6.53 ± 2.46, WS: 5.22 ± 3.69). Participants with DS were stopped more often than TD children (*t*_(__45__)_ = 3.374, *p* = 0.002), whereas there were no differences between participants with WS and TD children (*t*_(__44)_ = 1.354, *p* = 0.183) or between participants with WS and participants with DS (*t*_(__3__5)_ = 1.272, *p* = 0.212).

**TABLE 5:** Homing task.

	Homing Straight path S1	Homing Straight path S2	Homing Angled path S1	Homing Angled path S2
TD *n* =16	202 ± 71	184 ± 70	190 ± 73	203 ± 102
DS *n* =18	231 ± 94	164 ± 62	205 ± 85	225 ± 121
WS *n* =15	306 ± 122	244 ± 97	274 ± 83	266 ± 131
TD vs. DS	*t*_(__45)_ = 1.184 *p* = 0.243	*t*_(__45)_ = 1.010 *p* = 0.318	*t*_(__45)_ = 0.646 *p* = 0.522	*t*_(__45)_ = 0.668 *p* = 0.508
TD vs. WS	*t*_(__44)_ = 3.649 *p* = 0.001	*t*_(__44)_ = 2.450 *p* = 0.018	*t*_(__44)_ = 3.584 *p* = 0.001	*t*_(__44)_ = 1.813 *p* = 0.077
DS vs. WS	*t*_(__35)_ = 2.103 *p* = 0.043	*t*_(__35)_ = 3.024 *p* = 0.005	*t*_(__35)_ = 2.490 *p* = 0.018	*t*_(__35)_ = 0.980 *p* = 0.334

*Distance from home, in centimeters. Groupe average ± standard deviation.*

#### Initial Heading

The average initial heading of participants with WS, participants with DS and TD children did not differ from the ideal heading (Supplementary Material 2). Nevertheless, there were group differences in initial heading. In both sessions, the average initial heading of participants with WS deviated slightly to the right from the ideal heading, whereas the average initial heading of participants with DS deviated slightly to the left. In Session 1, the average initial heading of participants with DS differed from that of TD children. In both sessions, the average initial heading of participants with DS differed from that of participants with WS. Angular deviation (a measure of variability between participants within a group) did not differ between groups in either session (Supplementary Material 2). Accordingly, within-subject variability in initial heading (the length of the mean vector) did not differ between groups (*F*_(2__,62)_ = 0.746, *p* = 0.479) or between sessions (*F*_(2__,62)_ = 2.693, *p* = 0.106); there was no interaction between groups and sessions (*F*_(2,62__)_ = 0.676, *p* = 0.513).

#### Final Heading

The average final heading of participants with DS differed from the ideal heading in Session 1, but not in Session 2 (Supplementary Material 3). In contrast, the average final heading of participants with WS and TD children did not differ from the ideal heading in either session. The final heading differed between groups in Session 1, but not in Session 2. In Session 1, the average final heading of participants with WS deviated slightly to the right from the ideal heading, whereas the average final heading of participants with DS deviated slightly to the left. In Session 2, the average final heading did not differ between groups. Angular deviation (a measure of variability) differed between groups in both sessions (Supplementary Material 3). In Session 1, the angular deviation of TD children was smaller than the angular deviation of participants with DS and participants with WS; it did not differ between participants with DS and participants with WS. In Session 2, the angular deviation of participants with WS was greater than that of TD children, whereas it did not differ between participants with DS and the other groups. Within-subject variability in final heading (the length of the mean vector) did not differ between groups (*F*_(2,62)_ = 1.019, *p* = 0.367), or between sessions (*F*_(2,62)_ = 0.403, *p* = 0.528); there was no interaction between groups and sessions (*F*_(2,62)_ = 1.662, *p* = 0.198).

### Homing Task: Two-Legged Angled Outbound Paths

#### “Pass” or “Fail”

**FIGURE 3:**
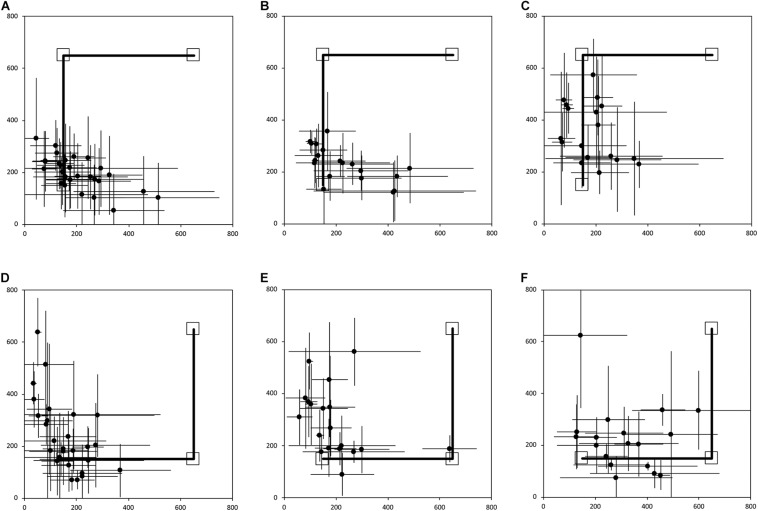
Average end location of individual participants’ return paths, following a two-legged 10 m angled outward journey in the egocentric homing task. The horizontal and vertical error bars represent the standard deviation for each individual across one session. The solid line indicates the outward angled path. The top right square indicates the starting point of the return path. The bottom left square indicates “home.” In Session 1, participants were guided by the left arm, and in Session 2 they were guided by the right arm. **(A)** TD, Session 1. **(B)** DS, Session 1. **(C)** WS, Session 1. **(D)** TD, Session 2. **(E)** DS, Session 2. **(F)** WS, Session 2. Room size: 800 cm × 800 cm.

Figure 3 shows the average end locations of TD children, participants with DS, and participants with WS who were asked to return “home” after being led blindfolded on a two-legged path of 10 m, with a 90° right turn after 5 m (Session 1) or a 90° left turn after 5 m (Session 2), thus ideally requiring a 135° right (Session 1) or 135° left (Session 2) turn at the end of the guided path, and a 7 m straight walk to return to home. For Session 1 (Figures 3A-C), 93% of TD children, 79% of participants with DS and 61% of participants with WS had an average end location in the quadrant of the room where the home was located (Tables 2-4). Thus, in Session 1, fewer participants with WS were considered to have passed the homing task than TD children (*X*^2^ = 7.017, *p* = 0.008). There was no difference in the number of participants with DS and the number of participants with WS (*X*^2^ = 1.408, *p* = 0.235) or TD children (*X*^2^ = 1.967, *p* = 0.161). For Session 2 (Figures 3D-F), 89% of TD children, 79% of participants with DS and 61% of participants with WS had an average end location in the quadrant of the room where the home was located (Tables 2-4). Thus, in Session 2, fewer participants with WS were considered to have passed the homing task than TD children (*X*^2^ = 5.112, *p* = 0.024). There was no difference in the number of participants with DS and the number of participants with WS (*X*^2^ = 1.408, *p* = 0.235) or TD children (*X*^2^ = 1.967, *p* = 0.161).

#### Average Distance From Home

There were differences between groups (*F*_(2, 62__)_ = 5.297, *p* = 0.008), but no difference between sessions (*F*_(2, 62__)_ = 0.259, *p* = 0.613) and no interaction between groups and sessions (*F*_(2, 62__)_ = 0.239, *p* = 0.788; Table 5). In Session 1, the distance between the end location and home was greater for participants with WS than for participants with DS or TD children. In Session 2, the distance between the end location and home did not differ between participants with WS and participants with DS or TD children. There were no differences between participants with DS and TD children in Session 1 or 2. It is important to note, also, that there were differences between groups in the number of times participants were stopped by the experimenter because they were approaching a wall (*F*_(2, 64)_ = 6.077, *p* = 0.004; out of 10 trials; TD: 4.14 ± 2.68 (average ± standard deviation); DS: 7.21 ± 2.92; WS: 5.89 ± 3.55). Participants with DS were stopped more often than TD children (*t*_(__45)_ = 3.719, *p* = 0.001). There was no difference between participants with WS and participants with DS (*t*_(__35)_ = 1.241, *p* = 0.223), and the difference between participants with WS and TD children failed to reach the predefined level of statistical significance (*t*_(__44)_ = 1.900, *p* = 0.064).

#### Initial Heading

The average heading after turning and walking one meter toward home did not differ from the ideal heading for participants with WS in either session (Supplementary Material 4). In contrast, the average initial heading of participants with DS and TD children differed from the ideal heading in both sessions. It deviated slightly to the left from the ideal heading for both groups in Session 1, whereas it deviated slightly to the right for both groups in Session 2. Accordingly, there were group differences in initial heading in both sessions. In addition, angular deviation (a measure of variability) was greater for the group of participants with DS than for the group of participants with WS in Session 2 (Supplementary Material 4). Within-subject variability in initial heading (the length of the mean vector) did not differ between groups (*F*_(2,62__)_ = 1.931, *p* = 0.154) or between sessions (*F*_(1,62__)_ = 0.754, *p* = 0.389); there was no interaction between groups and sessions (*F*_(2,62__)_ = 0.524, *p* = 0.595).

#### Final Heading

The average final heading did not differ from the ideal heading for any of the groups, for either session (Supplementary Material 5). Nevertheless, there were group differences in final heading in both sessions. In Session 1, the average final heading of the group of participants with WS deviated slightly to the right, and thus differed from the final heading of participants with DS and TD children, which both deviated slightly to the left. In Session 2, the average final heading of participants with WS deviated slightly to the left, and thus differed from the final heading of participants with DS and TD children, which both deviated slightly to the right. Angular deviation (a measure of variability) differed between groups in Session 1 but not in Session 2. In Session1, the angular deviation of participants with WS was greater than that of TD children; participants with DS did not differ from participants with WS or TD children. Accordingly, within-subject variability in final heading (the length of the mean vector) differed between groups (*F*_(2,62)_ = 3.858, *p* = 0.026); it did not differ between sessions (*F*_(1,62)_ = 0.247, *p* = 0.621) and there was no interaction between groups and sessions (*F*_(2,62)_ = 0.139, *p* = 0.871). The variability was greater for participants with WS, as compared to both TD children (*p* = 0.011) and participants with DS (*p* = 0.028). It did not differ between participants with DS and TD children (*p* = 0.876).

### Homing Task: Results Summary

We considered that a reasonably stringent criterion to define overall successful performance would require participants’ average final location to be in the home quadrant for at least 3 of the 4 sessions. A majority of TD children (96%) and participants with DS (84%), and a minority of participants with WS (44%) were capable of using path integration to build an egocentric spatial representation supporting homing behavior in absence of vision. Although the estimation that only 44% of the individuals with WS exhibited passing performance may not seem representative when considering that the percentage of individuals that passed on each of the four sessions ranged from 39 to 78%, it is critical to note that there was no consistency in the ability of individual participants to pass the different sessions. In other words, it was not always the same participants who succeeded in the different sessions, making an overall interpretation of greater success in individuals with WS misleading.

We next evaluated whether participants were capable of using path integration to build an allocentric spatial representation to take shortcuts, the hallmark of cognitive mapping abilities.

### Cognitive Mapping Task

#### “Pass” or “Fail”

**FIGURE 4:**
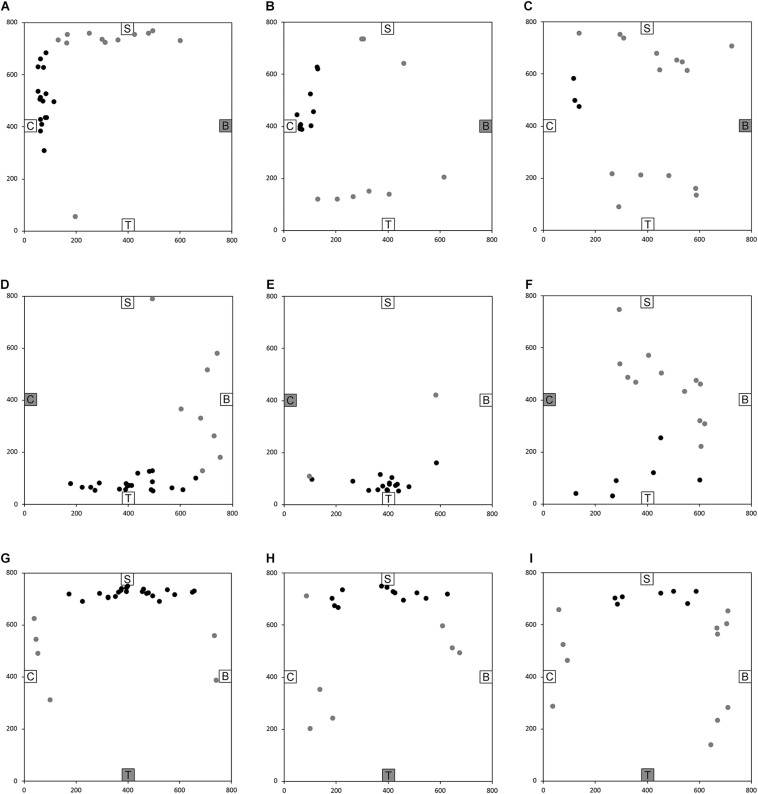
End location of participants in the novel path trials of the allocentric cognitive mapping task. **(A)** Bench to Chair, TD: 16/28 individuals ended in the quadrant of the room where the target object was located (black dot: in the correct quadrant; gray dot: in an incorrect quadrant). **(B)** Bench to Chair, DS: 10/19 individuals in the correct quadrant. **(C)** Bench to Chair, WS: 3/18 individuals in the correct quadrant. **(D)** Chair to Table, TD: 20/28 individuals in the correct quadrant. **(E)** Chair to Table, DS: 17/19 individuals in the correct quadrant. **(F)** Chair to Table, WS: 6/18 individuals in the correct quadrant. **(G)** Table to Shelf, TD: 22/28 individuals in the correct quadrant. **(H)** Table to Shelf, DS: 12/19 individuals in the correct quadrant. **(I)** Table to Shelf, WS: 7/18 individuals in the correct quadrant. Room size: 800 cm × 800 cm.

**FIGURE 5:**
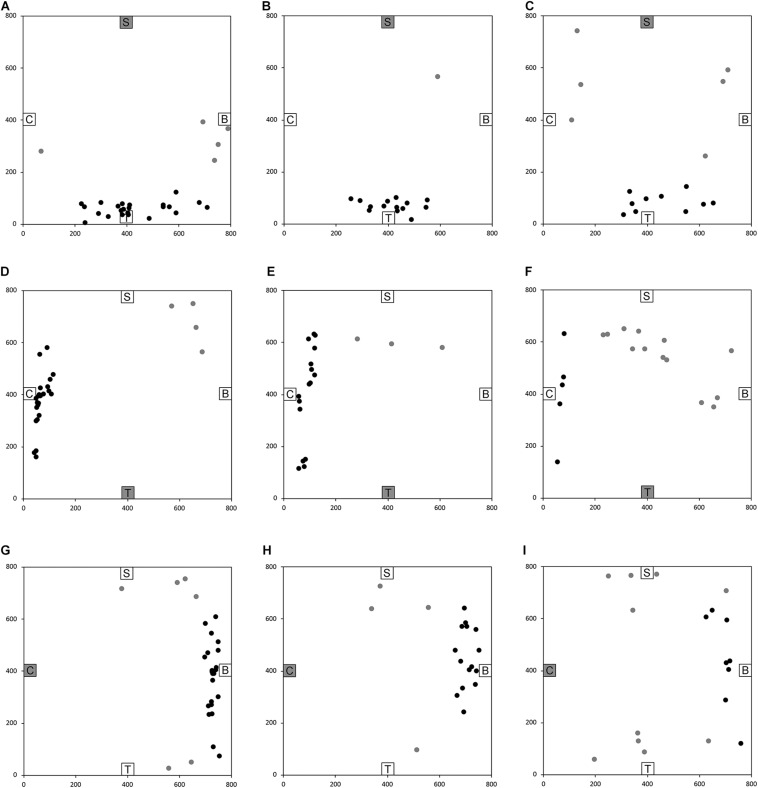
End location of participants in the novel reverse path trials of the allocentric cognitive mapping task. **(A)** Shelf to Table, TD: 23/28 individuals ended in the quadrant of the room where the target object was located (black dot: in the correct quadrant; gray dot: in an incorrect quadrant). **(B)** Shelf to Table, DS: 18/19 individuals in the correct quadrant. **(C)** Shelf to Table, WS: 12/18 individuals in the correct quadrant. **(D)** Table to Chair, TD: 24/28 individuals in the correct quadrant. **(E)** Table to Chair, DS: 16/19 individuals in the correct quadrant. **(F)** Table to Chair, WS: 5/18 individuals in the correct quadrant. **(G)** Chair to Bench, TD: 22/28 individuals in the correct quadrant. **(H)** Chair to Bench, DS: 15/19 individuals in the correct quadrant. **(I)** Chair to Bench, WS: 8/18 in the correct quadrant. Room size: 800 cm × 800 cm.

After having been blindfolded and guided along three selected paths between different objects, participants were asked to make six direct never-traveled trajectories between these objects, the first three being entirely novel paths and the last three being their reverse paths (Figure 1 and Supplementary Material 1). Figures 4, 5 show the individual end location, for each participant, for each trajectory. Between 57 and 86% of TD children exhibited passing performance by ending in the quadrant that contained the target object on each trial (Table 6). Between 53 and 95% of participants with DS exhibited passing performance by ending in the quadrant that contained the target object on each trial (Table 7). Between 17 and 67% of participants with WS exhibited passing performance by ending in the quadrant that contained the target object on each trial (Table 8). We considered that a reasonably stringent criterion to define overall successful performance in the cognitive mapping task would require participants to succeed on at least four of the six novel paths, including the two paths with a 45° angle (Chair to Table, Table to Chair). We reasoned that successful performance on the two paths with a 45° angle was necessary in order to infer the existence of a cognitive map, since successful performance on the other novel routes could be achieved by simply walking straight from the object at the beginning of the path, and that such a strategy might be adopted by participants that had not constructed a cognitive map of the spatial relationships between the four objects’ locations (Bostelmann et al., 2020). In contrast, however, failure to succeed on the straight paths should be considered as evidence against the ability to build a cognitive map. When applying this criterion, 64% of TD children, 74% of participants with DS, and 6% of participants with WS (Tables 6-8, respectively) demonstrated the ability to reliably travel to the target objects using novel paths, therefore demonstrating that they had built a cognitive map using path integration and could use this map to successfully navigate without vision.

**TABLE 6:** Individual performance of TD children for the never traveled trajectories of the cognitive mapping task.

Participant	Gender	Age (years)	Homing task	Allo task	Criterion	B-C	C-T	T-S	S-T	T-C	C-B
TD39	F	4.83	a2	/	Pass	Pass	Pass	Pass	Fail	Pass	Pass
TD160	M	4.83		/	Fail	Fail	Fail	Pass	Pass	Fail	Pass
TD167	M	4.83	s1	/	Fail	Fail	Pass	Pass	Pass	Fail	Fail
TD187	F	5.00		/	Pass	Pass	Pass	Pass	Pass	Pass	Pass
TD50	M	5.50	a2	Pass	Pass	Pass	Pass	Pass	Fail	Pass	Pass
TD53	F	5.50	a1	/	Pass	Fail	Pass	Pass	Pass	Pass	Pass
TD186	F	5.67		/	Fail	Fail	Fail	Fail	Fail	Pass	Fail
TD27	M	5.83		Pass	Fail	Pass	Pass	Fail	Fail	Pass	Fail
TD36	M	5.92	s1a2	Pass	Pass	Pass	Pass	Pass	Pass	Pass	Pass
TD34	F	6.67		Pass	Pass	Pass	Pass	Pass	Pass	Pass	Pass
TD35	M	6.67		Pass	Fail	Pass	Fail	Fail	Pass	Pass	Pass
TD48	F	6.67		Pass	Pass	Fail	Pass	Pass	Fail	Pass	Pass
TD141	F	6.67		/	Pass	Fail	Pass	Pass	Pass	Pass	Fail
TD42	M	6.92		Pass	Pass	Fail	Pass	Pass	Pass	Pass	Pass
TD49	F	7.08		Pass	Fail	Pass	Fail	Pass	Pass	Pass	Fail
TD143	F	7.08		/	Fail	Fail	Fail	Fail	Pass	Fail	Fail
TD31	F	7.17		Pass	Pass	Pass	Pass	Pass	Pass	Pass	Pass
TD25	M	7.25		Pass	Fail	Fail	Fail	Fail	Pass	Fail	Pass
TD191	M	7.50		/	Pass	Pass	Pass	Pass	Pass	Pass	Pass
TD26	F	7.92		Pass	Pass	Fail	Pass	Pass	Pass	Pass	Pass
TD29	F	7.92		Pass	Pass	Pass	Pass	Fail	Pass	Pass	Pass
TD52	M	8.08	a1	/	Pass	Pass	Pass	Pass	Pass	Pass	Pass
TD37	M	8.25		Pass	Pass	Pass	Pass	Pass	Pass	Pass	Pass
TD138	F	8.42		/	Pass	Pass	Pass	Pass	Pass	Pass	Pass
TD28	F	8.58		Pass	Pass	Fail	Pass	Pass	Pass	Pass	Pass
TD30	F	8.92		Pass	Fail	Pass	Fail	Pass	Pass	Pass	Pass
TD142	M	9.17		/	Pass	Pass	Pass	Pass	Pass	Pass	Pass
TD43	M	9.67		Pass	Fail	Fail	Fail	Pass	Pass	Pass	Pass
			Pass	16	18	16	20	22	23	24	22
			Fail	0	10	12	8	6	5	4	6
			% Pass	100%	64%	57%	71%	79%	82%	86%	79%

*B-C, Bench to Chair; C-T, Chair to Table; T-S, Table to Shelf; S-T, Shelf to Table; T-C, Table to Chair; C-B, Chair to Bench; Homing task, the letter/number indicates sessions in which the participant was considered to have failed; s1, straight session 1; s2, straight session 2; a1, angled session 1; a2, angled session 2. Allo task, performance of participants who were also tested in the open-field allocentric spatial learning task (Bostelmann et al., 2017, 2018): Pass, succeeded at the task; Fail, failed the task; /, not tested. Pass, average final location within the target quadrant; Fail, average final location not within the target quadrant. Criterion: 4 paths passed, including the C-T and the T-C paths.*

**TABLE 7:** Individual performance of participants with DS for the never traveled trajectories of the cognitive mapping task.

Participant	Gender	M. A. (years)	Homing task	Allo task	Criterion	B-C	C-T	T-S	S-T	T-C	C-B
DS19	M	4.67	s1a2	Pass	Pass	Pass	Pass	Fail	Pass	Pass	Pass
DS6	M	4.75	s1	Pass	Pass	Pass	Pass	Pass	Pass	Pass	Pass
DS8	F	4.75	s1a2	Pass	Fail	Fail	Fail	Fail	Pass	Pass	Fail
DS1	M	4.83	a2	Fail	Fail	Fail	Pass	Pass	Pass	Fail	Pass
DS5	F	5.00	s1	Pass	Pass	Pass	Pass	Pass	Pass	Pass	Pass
DS13	M	5.00	a2	Pass	Fail	Pass	Pass	Fail	Pass	Fail	Pass
DS12	M	5.08		Fail	Pass	Pass	Pass	Pass	Pass	Pass	Pass
DS30	M	5.25		Fail	Pass	Pass	Pass	Pass	Pass	Pass	Pass
DS24	M	5.29		Pass	Pass	Fail	Pass	Pass	Pass	Pass	Pass
DS7	M	5.33		Fail	Pass	Pass	Pass	Pass	Pass	Pass	Pass
DS15	F	5.33		/	Pass	Pass	Pass	Pass	Pass	Pass	Pass
DS23	M	5.58	a1	Pass	Fail	Fail	Pass	Fail	Pass	Pass	Fail
DS25	F	5.88	a1	Pass	Pass	Fail	Pass	Pass	Pass	Pass	Pass
DS27	F	6.00	a1	Pass	Pass	Fail	Pass	Pass	Pass	Pass	Pass
DS26	F	6.21	s1s2	Pass	Fail	Fail	Fail	Fail	Fail	Fail	Fail
DS2	F	6.67		Pass	Pass	Pass	Pass	Fail	Pass	Pass	Pass
DS4	F	6.67		Pass	Pass	Fail	Pass	Pass	Pass	Pass	Pass
DS22	M	6.67		Pass	Pass	Fail	Pass	Pass	Pass	Pass	Pass
DS21	F	6.96	a1	Pass	Pass	Pass	Pass	Fail	Pass	Pass	Fail
			Pass	14	14	10	17	12	18	16	15
			Fail	4	5	9	2	7	1	3	4
			% Pass	78%	74%	53%	89%	63%	95%	84%	79%

*B-C, Bench to Chair; C-T, Chair to Table; T-S, Table to Shelf; S-T, Shelf to Table; T-C, Table to Chair; C-B, Chair to Bench; Homing task, the letter/number indicates sessions in which the participant was considered to have failed; s1, straight session 1; s2, straight session 2; a1, angled session 1; a2, angled session 2. Allo task, performance of participants who were also tested in the open-field allocentric spatial learning task (Bostelmann et al., 2017, 2018): Pass, succeeded at the task; Fail, failed the task; /, not tested. Pass, average final location within the target quadrant; Fail, average final location not within the target quadrant. Criterion: 4 paths passed, including the C-T and the T-C paths.*

**TABLE 8:** Individual performance of participants with WS for the never traveled trajectories of the cognitive mapping task.

Participant	Gender	M. A. (years)	Homing task	Allo task	Criterion	B-C	C-T	T-S	S-T	T-C	C-B
WS13	F	4.42	s1	Fail	Fail	Fail	Fail	Pass	Pass	Fail	Pass
WS2	M	4.75		Fail	Fail	Pass	Fail	Pass	Fail	Fail	Fail
WS15	M	4.75	s1	Pass	Fail	Pass	Fail	Fail	Pass	Fail	Pass
WS22	F	5.00	a1a2	/	Fail	Fail	Pass	Pass	Pass	Fail	Fail
WS3	M	5.33		Fail	Fail	Fail	Fail	Fail	Pass	Fail	Fail
WS5	F	5.33	s1a2	Fail	Fail	Fail	Fail	Fail	Pass	Pass	Fail
WS18	F	5.54	s1	Fail	Fail	Fail	Pass	Pass	Fail	Fail	Fail
WS10	F	5.92	s1s2a2	Fail	Fail	Fail	Fail	Pass	Pass	Fail	Pass
WS17	M	6.00	s1a1	Fail	Fail	Fail	Pass	Pass	Fail	Fail	Pass
WS7	M	6.21	s1a1a2	Fail	Fail	Fail	Fail	Fail	Fail	Pass	Fail
WS8	M	6.21	s1a1	Fail	Fail	Fail	Fail	Fail	Pass	Pass	Fail
WS4	M	6.67	s1a1a2	Fail	Fail	Fail	Pass	Fail	Pass	Pass	Fail
WS9	F	7.00	a2	Fail	Fail	Fail	Fail	Fail	Pass	Fail	Pass
WS1	F	7.08	s2a2	Fail	Fail	Fail	Fail	Fail	Fail	Fail	Fail
WS12	M	7.08		Pass	Pass	Pass	Pass	Fail	Pass	Pass	Pass
WS16	M	7.50	s2	Pass	Fail	Fail	Fail	Fail	Fail	Fail	Fail
WS20	M	-	s1s2a1	/	Fail	Fail	Pass	Fail	Pass	Fail	Pass
WS21	F	-	s1a1	/	Fail	Fail	Fail	Pass	Pass	Fail	Pass
			Pass	3	1	3	6	7	12	5	8
			Fail	12	17	15	12	11	6	13	10
			% Pass	20%	6%	17%	33%	39%	67%	28%	44%

*B-C, Bench to Chair; C-T, Chair to Table; T-S, Table to Shelf; S-T, Shelf to Table; T-C, Table to Chair; C-B, Chair to Bench; Homing task, the letter/number indicates sessions in which the participant was considered to have failed; s1, straight session 1; s2, straight session 2; a1, angled session 1; a2, angled session 2. Allo task, performance of participants who were also tested in the open-field allocentric spatial learning task (Bostelmann et al., 2017, 2018): Pass, succeeded at the task; Fail, failed the task; /, not tested. Pass, average final location within the target quadrant; Fail, average final location not within the target quadrant. Criterion: 4 paths passed, including the C-T and the T-C paths.*

#### Average Distance From the Target

There were differences between groups in the distance between the participant’s end location and the target object (*F*_(2,62__)_ = 14.151, *p* < 0.001), differences between paths (*F*_(__5,310__)_ = 3.962, *p* = 0.002), and no interaction between groups and paths (*F*_(__10,310__)_ = 1.170, *p* = 0.310; Tables 9-10). The distance from the target was greater for participants with WS than for participants with DS for all six paths, except for the path between the table and the shelf. The distance from the target was greater for participants with WS than for TD children for all six paths. There were no differences between participants with DS and TD children except for the path between the chair and the table, for which the distance from the target of participants with DS was actually shorter than that of TD children.

**TABLE 9:** Cognitive mapping task. Distance from home, in centimeters.

	Bench to Chair	Chair to Table	Table to Shelf
TD *n* =16	264 ± 189	212 ± 195	175 ± 158
DS *n* =18	240 ± 188	101 ± 119	234 ± 179
WS *n* =15	425 ± 166	353 ± 164	320 ± 188
TD vs. DS	*t*_(__45)_ = 0.432 *p* = 0.668	*t*_(__45)_ = 2.206 *p* = 0.033	*t*_(__45)_ = 1.186 *p* = 0.242
TD vs. WS	*t*_(__44)_ = 2.966 *p* = 0.005	*t*_(__44)_ = 2.541 *p* = 0.015	*t*_(__44)_ = 2.813 *p* = 0.007
DS vs. WS	*t*_(__35)_ = 3.176 *p* = 0.003	*t*_(__35)_ = 5.365 *p* < 0.001	*t*_(__35)_ = 1.425 *p* = 0.163

*Groupe average ± standard deviation.*

**TABLE 10:** Cognitive mapping task.

	Shelf to Table	Table to Chair	Chair to Bench
TD *n* =16	169 ± 141	163 ± 218	172 ± 141
DS *n* =18	144 ± 120	203 ± 145	179 ± 145
WS *n* =15	285 ± 220	365 ± 192	334 ± 196
TD vs. DS	*t*_(__45)_ = 0.622 *p* = 0.537	*t*_(__45)_ = 0.704 *p* = 0.485	*t*_(__45)_ = 0.172 *p* = 0.864
TD vs. WS	*t*_(__44)_ = 2.179 *p* = 0.035	*t*_(__44)_ = 3.216 *p* = 0.002	*t*_(__44)_ = 3.273 *p* = 0.002
DS vs. WS	*t*_(__35)_ = 2.426 *p* = 0.021	*t*_(__35)_ = 2.909 *p* = 0.006	*t*_(__35)_ = 2.749 *p* = 0.009

*Distance from home, in centimeters. Groupe average ± standard deviation.*

#### Initial Heading

For all three groups, the average initial heading did not differ from the ideal heading, except for the two paths requiring a 45° angle (Supplementary Material 6). Similarly, there were no differences between groups in initial heading, except for the two paths requiring a 45° angle. For these two paths, participants with WS deviated more from the ideal heading and tended to orient straight ahead, whereas participants with DS or TD children initiated their walk with an angle; there was no difference in the average initial heading between participants with DS and TD children. For these two paths, the angular deviation (a measure of variability) of participants with WS was greater than that of participants with DS and TD children; there was no difference in angular deviation between participants with DS and TD children.

#### Final Heading

The average final heading differed from the ideal heading for TD children for the first novel path, and for participants with WS for the two paths requiring a 45° angle (Supplementary Material 7). The average final heading of participants with WS differed from those of participants with DS and TD children for the two paths with a 45° turn. Angular deviation (a measure of variability) was greater for participants with WS than for participants with DS and TD children for all paths, except for the path from the table to the shelf for which the difference between participants with WS and participants with DS was not statistically significant. There was no difference in angular deviation between participants with DS and TD children.

One important difference between initial and final headings must be noted. For all three groups the average initial heading differed from the ideal heading for the two paths requiring a 45° turn. However, at the end of their trajectory, only the average final heading of the group of participants with WS differed from the ideal heading. In contrast, the average final heading of participants with DS and that of TD children no longer differed from the ideal heading. These findings suggest that participants with DS and TD children may have solved the 45° angle paths by first walking somewhat straight (albeit less straight than participants with WS), and then by angling toward the target object at some point after one meter.

### Cognitive Mapping Task: Results Summary

Altogether, our findings showed that the average initial and final headings of participants with WS differed from the ideal heading for the two 45° angle paths. Moreover, for these two critical paths participants with WS were overall less accurate and more variable than participants with DS and TD children. Altogether, individual data and group analyses indicate that a majority of participants with DS (74%), a proportion slightly higher than that of TD children (64%; the difference was not statistically significant), exhibited the ability to use path integration to build a cognitive map of the environment in absence of visual information. In contrast, only one participant with WS was able to use path integration to build a cognitive map, whereas the vast majority (94%) were not, even though almost half of the participants with WS (44%) were able to use path integration to support homing behavior.

## Discussion

This study aimed to characterize the capacity of individuals with DS and individuals with WS to use path integration to build egocentric and allocentric spatial representations to navigate in a real-world environment without vision. We found that 84% of participants with DS could use path integration to return to a home base and 74% of participants with DS could build a cognitive map. In contrast, only 44% of participants with WS could use path integration to return to a home base consistently, and only 6% of participants with WS could build a cognitive map. Our study thus revealed that in a real-world laboratory setting, individuals with DS exhibit cognitive mapping abilities that are similar to those of TD children within the same mental age range, whereas individuals with WS are comparatively impaired. These findings are consistent with previous findings suggesting a relative preservation of the ability to create and use low-resolution allocentric spatial representations in DS (Banta Lavenex et al., 2015; Bostelmann et al., 2018), and significant impairments in WS (Bostelmann et al., 2017).

### Comparison With Previous Studies in Individuals With DS

#### Egocentric Tasks in the Real-World

Bostelmann et al. (2018) tested the ability of individuals with DS and TD children to solve a spatial response-learning task in a 4 m × 4 m open-field arena. In that task, the reward was hidden in one of four possible locations, which alternated between the left and right sides of the arena on each trial. It was therefore not in the same location with respect to the environment, but instead was always found opposite from where the participant entered the arena. Optimal performance in this response-learning task requires not only the ability to encode the reward location in a viewpoint-dependent manner and always walk across the arena to find the reward along the middle of the opposite wall, but also the ability to ignore contradictory allocentric strategies that would lead the participant to return to the location where the reward was found on the preceding trial. Interestingly, whereas 95% of TD children exhibited optimal performance on an allocentric spatial memory task in the same arena (see below), only 16% succeeded on the response-learning task. In fact, most TD children persisted in employing an allocentric strategy and returning to the location where the reward was found on the preceding trial. In contrast, 56% of participants with DS succeeded on the response-learning task, demonstrating that they were better at ignoring conflicting allocentric strategies. In the current study, homing behavior could be supported by an egocentric spatial representation of the individual’s position with respect to their starting position on the outbound journey, which did not conflict with any other spatial representation or strategy to guide behavior. We found that 84% of participants with DS could return to home consistently, only slightly fewer than the number of TD children (96%; the difference was not statistically significant). These results demonstrating relatively preserved egocentric capacities in DS are also in agreement with findings from a recent study showing that individuals with DS performed as well as MA-matched TD children in reproducing an egocentric 1- to 7-step route (consisting of sequential moves forward, right or left) on a 4 × 4 floor matrix comprising 16 squares of 50 cm × 50 cm separated by 10 cm gaps, after either studying a map of the route or observing an experimenter take the same route (Meneghetti et al., 2020). Specifically, individuals with DS were able to reproduce an average of 3.00 steps after visualizing the trajectory on a map, while MA-matched TD children reproduced an average of 3.53 steps. After observing an experimenter walk along the same path, individuals with DS reproduced an average of 3.50 steps, while TD children reproduced an average of 4.23 steps. Interestingly, both individuals with DS and TD children performed better after observing the experimenter walking the route (real-world correspondence) than after studying the route on a map (transfer from a schematic representation to the real world).

#### Egocentric Tasks in Virtual Reality

Our results are partially consistent with studies investigating egocentric route learning in virtual environments, which have found that the majority of individuals with DS can learn the routes, yet their performance is not always comparable to that of MA-matched TD children, and the specific learning strategies used by the different groups of participants may differ (Purser et al., 2012; Courbois et al., 2013; Davis et al., 2014; Farran et al., 2015; N’Kaoua et al., 2019). Altogether, these findings suggested that participants with DS may pay less attention to environmental landmarks or have more difficulty in associating those landmarks with specific spatial locations or behaviors, and are thus more likely to rely on a sequence of directional changes to learn routes. In contrast, TD children may benefit from the congruence of the two strategies, relying both on the sequence of directional changes and their consistency with environmental landmarks at choice points or along the path (Purser et al., 2015). In the present study, since visual information could not be used to support navigational strategies and thus facilitate performance in TD children, we found similar performance for participants with DS and MA-matched TD children.

#### Allocentric Tasks in the Real-World

Our findings that 74% of participants with DS were capable of using path integration to build a cognitive map of the four objects’ locations in absence of visual information are very similar to previous findings from an open-field allocentric spatial memory task showing that 78% of participants with DS were able to learn and remember the location of one reward amongst four identical locations in the presence of visual information (Table 11) (Bostelmann et al., 2018). Importantly, a majority of our participants in all three groups (DS, WS and TD children) were also tested previously in this open-field task, allowing us to compare the performance of these individuals across these two allocentric tasks theoretically subserved by the same neural substrates. For both tasks, in order to succeed participants had to create a cognitive map of the environment using path integration, but in one condition in the presence of vision (open-field arena), and in the other in the absence of vision (cognitive mapping task). Of the sixteen TD children who were tested on both tasks, all passed the open-field allocentric task, whereas 10 passed and 6 failed the cognitive mapping task (this proportion was similar to that of the TD children who participated only in the cognitive mapping study). Note that the only TD child who failed the open-field task with one location (a 3.5-year-old boy) refused to walk with the sleeping mask covering his eyes and was therefore not tested in the present study. Thus, fewer TD children passed the cognitive mapping task than the open-field task. However, performance was not correlated with age, suggesting that children who failed may have been dismissive or inattentive during the learning phase (Bostelmann et al., 2020). Of the 18 participants with DS who were tested on both tasks, 14 passed the open-field task, whereas 13 passed the cognitive mapping task. Surprisingly, of the 13 participants with DS who passed the cognitive mapping task, three failed the open-field task with one location, a finding which may highlight poor comprehension of the goals of the task or an inability to inhibit egocentric responding in the open-field task.

**TABLE 11:** Number of participants who passed or failed the open-field allocentric spatial memory task with one location and with vision (Bostelmann et al., 2017, 2018) and the cognitive mapping task with four objects in the absence of vision (current study).

	Pass Allo & Pass CogMap	Pass Allo & Fail CogMap	Fail Allo & Pass CogMap	Fail Allo & Fail CogMap
TD *n* =16	10	6	0	0
DS *n* =18	10	4	3	1
WS *n* =15	1	2	0	12

In sum, both theoretical and empirical evidence support the view that performance in both tasks depends on the ability to form and use a low-resolution allocentric spatial representation of the environment. Our data show that this ability is largely preserved in individuals with DS. It is important to note, however, that the ability to form and use high-resolution allocentric spatial representations of the environment has been shown to be impaired in a majority of individuals with DS (Banta Lavenex et al., 2015).

#### Allocentric Task in Virtual Reality

Although several studies reported an impaired ability of individuals with DS to demonstrate allocentric spatial capacities or configural knowledge of landmark locations using virtual reality paradigms, no study has provided unequivocal evidence of a specific impairment as compared to MA-matched TD children (Purser et al., 2012; Courbois et al., 2013; Farran et al., 2015; N’Kaoua et al., 2019). The main reason is the fact that none of these studies actually demonstrated that the TD children relied on an allocentric or configural spatial representation to solve these tasks, thus making comparisons with individuals with DS uninformative. In another example, Pennington et al. (2003) tested individuals with DS and MA-matched TD children on a virtual Morris water maze task. During a probe trial in absence of the platform, participants with DS spent on average less time searching in the quadrant of the platform than TD children. However, other factors such as motivation or the drive to explore other parts of the environment to look for the platform may also influence the time spent searching in the target quadrant. Indeed, in a subsequent study, Edgin et al. (2010) failed to reveal any difference between participants with DS and MA-matched TD children, thus raising doubts about a global impairment of allocentric spatial capacities in individuals with DS. Finally, Toffalini et al. (2018) evaluated the ability of individuals with DS and MA-matched TD children to learn the locations of five local landmarks distributed at the four corners and along one of the walls of a square area. Although some subtle differences were reported in the performance of the two groups in different learning conditions, statistical analyses suggested that participants with DS performed as well as TD children when asked to place the landmarks at their approximate locations on a layout of the environment.

In sum, previous studies carried out in virtual reality have not provided reliable evidence regarding the ability of individuals with DS to build an allocentric spatial representation of their environment. In contrast, the current study has shown unequivocally that 74% of participants with DS were able to build a cognitive map in absence of visual information. These findings are consistent with previous findings showing that 78% of participants with DS were able to learn and remember the location of one reward amongst four identical locations in presence of visual information (Bostelmann et al., 2018).

### Comparison With Previous Studies in WS

#### Egocentric Tasks in the Real-World

Bostelmann et al. (2017) previously tested the ability of individuals with WS to solve the egocentric response-learning task and the allocentric place learning task as described above for individuals with DS and TD children (Bostelmann et al., 2018). Again, whereas 95% of TD children exhibited optimal performance on the allocentric spatial memory task, only 16% passed the response-learning task. In contrast, 72% of participants with WS succeeded on the response-learning task, thus exhibiting superior and even facilitated egocentric response-learning as compared to TD children. In the current study, however, only 44% of participants with WS were able to use an egocentric strategy to consistently return to the starting point of the outbound journey. It is possible that the differing performance of individuals with WS on these two different egocentric tasks can be explained by the presence of visual landmarks or beacons that can be used in combination with idiothetic information to confirm the target location. Indeed, in the egocentric response-learning task participants can see the potential reward locations that when combined with idiothetic cues allow them to encode the reward location in a viewpoint-dependent manner and thus always walk across the arena to choose the visible reward location along the middle of the opposite wall. These findings indicate that the performance of participants with WS is overall less accurate and more variable than that of MA-matched TD children and participants with DS when using egocentric representations constructed from self-generated motion information alone.

Accordingly, individuals with WS have been shown to be able to learn a new 1 km long route including 20 choice points (left, right, straight ahead) through an unfamiliar environment, although they performed less well than MA-matched TD children (Farran et al., 2010). However, the performance of participants with WS improved if they were given verbal instructions including directional information and information about features along the routes, including the highlighting of four major landmarks to remember for future use, and repeated experience walking the route. These findings suggest that individuals with WS benefit from verbal memory and the presence of visual landmarks along the path, and may therefore rely on sequential egocentric responses combined with viewpoint-matching of a series of landmarks in order to learn a route in a real-world environment.

#### Egocentric Tasks in Virtual Reality

Studies carried out in virtual reality also suggest that a majority of individuals with WS are capable of route learning using visual landmarks located along the path (Broadbent et al., 2014, 2015; Farran et al., 2015). In a study using a design similar to the one described above for individuals with DS (Courbois et al., 2013), Farran et al. (2015) reported that about two thirds of individuals with WS can learn at least one of two different routes requiring four changes of direction. Using a differently shaped virtual environment, Broadbent et al. (2015) showed that individuals with WS exhibited a reliance on visual landmarks for route-learning and failed to learn a route containing 6 changes in direction that did not contain landmarks. These findings are consistent with those reported by Broadbent et al. (2014) for the learning of a route comprising 4 directional changes in a cross-maze virtual environment. Thus, in contrast to what has been shown for individuals with DS, who do not benefit from the presence of environmental landmarks but can learn a sequence of directional changes, individuals with WS appear to rely more heavily on local environmental landmarks.

#### Allocentric Tasks in the Real-World

Our findings that only one of 18 participants with WS was capable of using path integration to build a cognitive map in the absence of visual information are consistent with previous findings in the open-field allocentric spatial memory task, which showed that only 17% of participants with WS were capable of using a low-resolution allocentric spatial representation to learn and remember the location of one reward amongst four possible locations in the presence of visual information (Bostelmann et al., 2017). Of the participants tested in the current study, sixteen TD children and 15 participants with WS were also tested in the open-field allocentric spatial memory task (Table 11). As described above, 100% of the TD children passed the open-field task, whereas 64% of these TD children passed the cognitive mapping task and 36% failed. In contrast, only 20% of participants with WS passed the open-field task (3 of 15), and only one of these individuals passed the cognitive mapping task, whereas the other two failed. Thus, as discussed above, theoretical and empirical evidence suggest that performance on both tasks depends on the ability to form and use a low-resolution allocentric spatial representation of the environment. Our findings show that the vast majority of participants with WS are unable to solve either task.

In agreement with our findings, Farran et al. (2010) showed that individuals with WS learned a route through a natural environment, and that their route knowledge was improved by verbal coding of the route, and by walking it more than once (Farran et al., 2010). However, in contrast to MA-matched TD children, individuals with WS did not appear to learn the spatial relationships between environmental landmarks, as shown by their inability to point accurately in the direction of several landmarks from different points along the route. As discussed previously (Bostelmann et al., 2017), although other studies employing real-world paradigms have suggested deficits in allocentric spatial processing in individuals with WS, because success in these paradigms was also dependent on other cognitive processes [i.e., the ability to understand complex verbal instructions, mental rotation and working memory (Nardini et al., 2008; Mandolesi et al., 2009; Foti et al., 2011)] or could be solved using egocentric coding of multiple visible locations (Smith et al., 2009; Foti et al., 2011), it was not clear whether allocentric spatial learning per se was impacted in WS. In contrast, our current and previous findings (Bostelmann et al., 2017) provide unequivocal evidence that allocentric spatial processes are severely impaired, if not abolished, in a large majority of individuals with WS.

#### Allocentric Tasks in Virtual Reality

As discussed above for individuals with DS, although a few studies have reported an impaired ability of individuals with WS to demonstrate allocentric spatial capacities or configural knowledge of landmark locations using virtual reality paradigms (Broadbent et al., 2015; Farran et al., 2015), they did not provide unequivocal evidence of a specific impairment as compared to MA-matched TD children. The main reason was that these paradigms did not conclusively demonstrate that TD children actually relied on an allocentric or configural spatial representation to solve these tasks either. Further confirmation of this conclusion is a study by Broadbent et al. (2014) which showed that less than 50% of 10-year-old TD children may have used an allocentric strategy to solve a cross-maze task, and only between 20 and 30% of TD children between 5 and 8 years of age, which corresponds to the mental age of individuals with WS, may have solved the task using an allocentric strategy. Such poor performance by TD children makes comparisons of the performance of individuals with WS relatively uninformative.

## Conclusion

Our study revealed that in a real-world laboratory setting, individuals with DS exhibit homing and cognitive mapping abilities similar to those of TD children with similar mental ages. These results are consistent with previous findings suggesting a preservation of the neural circuits subserving the creation and use of low-resolution egocentric and allocentric spatial representations in DS. In contrast, individuals with WS are severely impaired in their ability to build cognitive maps, and although many are impaired in their ability to home without vision, more individuals may be capable of successful route finding in the presence of visual landmarks. These findings are consistent with previous findings suggesting abnormalities of the neural circuits subserving the creation of egocentric and allocentric spatial representations in WS.

Thus, although individuals with DS and WS have similar mental ages, they exhibit distinct spatial cognitive profiles that should be considered carefully when designing training paradigms to improve navigational capacities that can lead to greater autonomy, self-confidence and social inclusion. Specifically, preserved capacities should be targeted to develop syndrome-specific navigational strategies. Since individuals with WS are essentially unable to build cognitive maps of their environment, they should not be expected or trained to use cognitive mapping strategies, since these strategies are unlikely to be successful. In contrast, individuals with WS can be encouraged to memorize sequences of directional changes to learn an itinerary and may benefit from the presence of environmental landmarks to learn a route from point A to point B. For individuals with DS, due to an overall weakness in their working memory capacities, navigation training should not include strategies that rely heavily on the memorization of multiple sequences of directional changes or environmental landmarks. Because individuals with DS can build low-resolution cognitive maps they should be encouraged to have confidence in their overall sense of direction, and to use cognitive mapping strategies when navigating.

## Data Availability Statement

The raw data supporting the conclusions of this article will be made available by the authors, without undue reservation.

## Ethics Statement

The studies involving human participants were reviewed and approved by the Commission Cantonale d’Éthique de la Recherche sur l’Être Humain, Vaud, Switzerland. Written informed consent to participate in this study was provided by the participants’ legal guardian/next of kin.

## Author Contributions

PBL and PL were responsible for the conception and design of the work, acquisition, analysis and interpretation of the data, and writing of the manuscript. MB was responsible for the design of the work, acquisition, analysis and interpretation of the data, and drafting of the manuscript. PR was responsible for data acquisition and drafting of the manuscript. AR, FC, and DM were responsible for data acquisition. SV was responsible for conception of the work and drafting of the manuscript. All authors approved the final version of the manuscript.

## Conflict of Interest

The authors declare that the research was conducted in the absence of any commercial or financial relationships that could be construed as a potential conflict of interest.
