# Supplementary material for: Path Integration and Cognitive Mapping Capacities in Down and Williams Syndromes
Source: Front Psychol. 2020 Dec 11;11:571394. doi: 10.3389/fpsyg.2020.571394 (PMC7759488; doi:10.3389/fpsyg.2020.571394)
Supplement: Supplementary file 1 [file Data_Sheet_1.docx]

**Supplementary Material 1**: Schematic representation of the experimental design and sequence of trajectories performed by participants in the homing and cognitive mapping tasks.

| 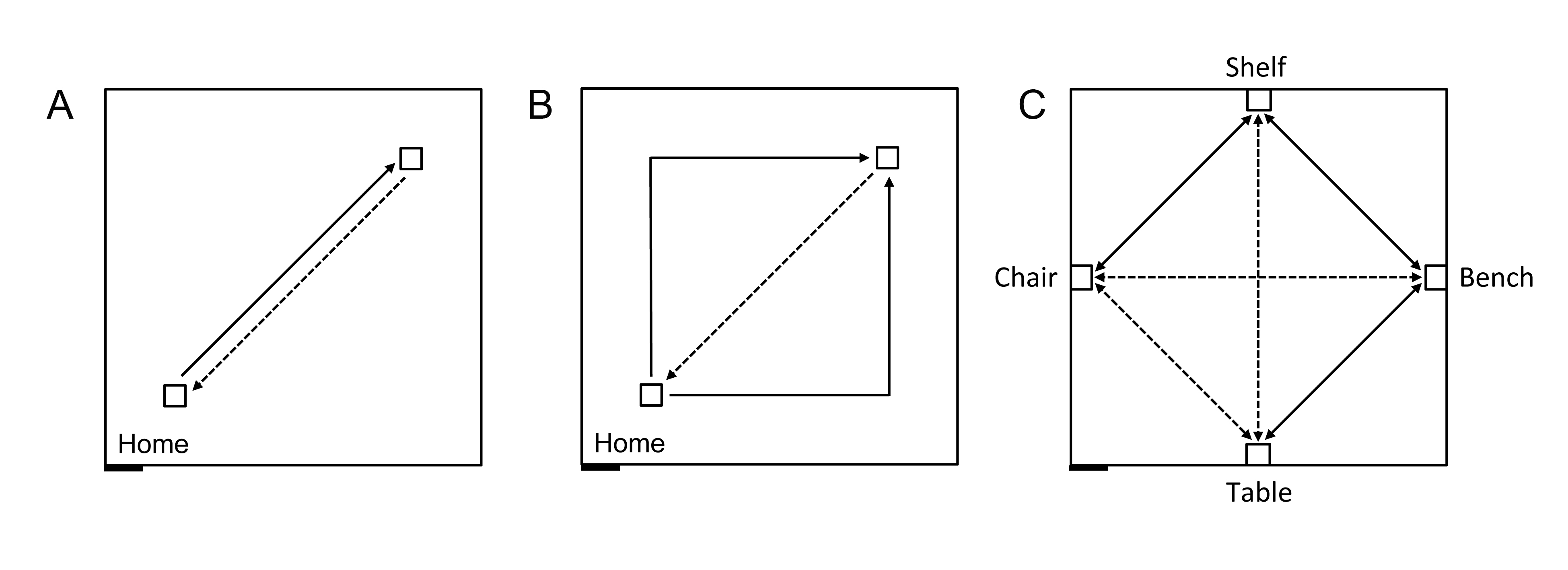 |
| --- |
| Figure 1.1 Schematic representation of the experimental design, carried out in an 8 m x 8 m testing room. The black rectangle at the bottom left represents the entry door to the room. Solid lines indicate guided trajectories; dashed lines indicate direct paths that participants were verbally requested to make. A. Homing task, straight paths: 7 m straight line guided trajectory, 7 m return path. B. Homing task, angled paths: 10 m angular guided trajectory with a right or left turn (5 m + 5 m), and 7 m return path. C. Cognitive mapping task: Guided routes (solid) and novel routes (dashed) between four objects, without vision. The paths between the bench and the chair, and between the table and the shelf were 7 m long; the other paths were 5 m long. |

| **Table 1.1** Sequence of trajectories performed by participants in the cognitive mapping task. | |
| --- | --- |
|  | **Route learning paradigm** |
| Learning Phase | Bench to shelf round-trip guided 2 x |
|  | Bench to shelf round-trip alone 1 x |
|  | Bench to shelf round-trip guided 2 x |
|  | Bench to shelf round-trip alone 2 x |
|  | Bench to shelf 1-way alone 1 x |
|  | Shelf to chair round-trip guided 2 x |
|  | Shelf to chair round-trip alone 1 x |
|  | Shelf to chair round-trip guided 2 x |
|  | Shelf to chair round-trip alone 2 x |
|  | Shelf to bench 1-way alone 1 x |
|  | Bench to table round-trip guided 2 x |
|  | Bench to table round-trip alone 1 x |
|  | Bench to table round-trip guided 2 x |
|  | Bench to table round-trip alone 2 x |
| Testing Phase | Bench to chair alone 1 x |
|  | Chair to table alone 1 x |
|  | Table to shelf alone 1 x |
|  | Shelf to table alone 1 x |
|  | Table to chair alone 1 x |
|  | Chair to bench alone 1 x |

**Supplementary Material 2.** Homing task - Straight outward paths –Initial heading

| 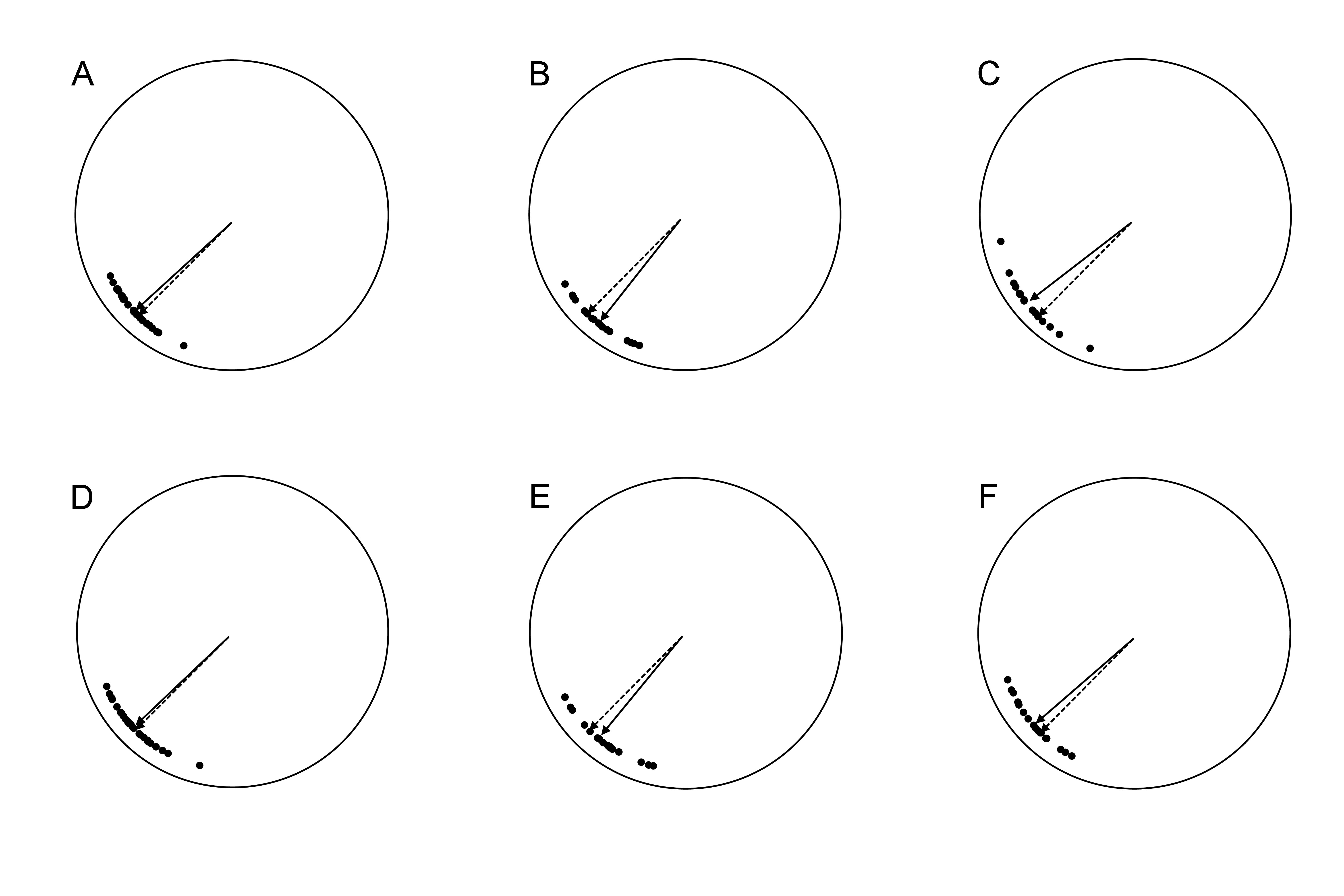 |
| --- |
| **Figure 2.1** Average (solid-line arrow) and individual (dots) walking directions of participants after one meter along the return path, following a straight 7 m outward journey in the homing task. A. TD, Session 1. B. DS, Session 1. C. WS, Session 1. D. TD, Session 2. E. DS, Session 2. F. WS, Session 2. Dashed-line arrow: perfect home direction. |

| **Table 2.1 Within-group results:** Homing task - Straight outward paths - Initial heading | | | | | | | | | | | | |
| --- | --- | --- | --- | --- | --- | --- | --- | --- | --- | --- | --- | --- |
|  | TD | | | | DS | | | | WS | | | |
| Path | Initial Head. |  | 99%  C.I. | Ang.  Dev. | Initial Head. |  | 99%  C.I. | Ang.  Dev. | Initial Head. |  | 99%  C.I. | Ang.  Dev. |
| Session 1 | 2.31° | < | 5.41° | 10.32° | 6.32° | < | 7.80° | 11.83° | 7.56° | < | 9.44° | 13.77° |
| Session 2 | 1.47° | < | 6.25° | 11.87° | 5.55° | < | 10.15° | 15.21° | 4.43° | < | 8.25° | 12.11° |

| **Table 2.2 Between groups comparisons:** Homing task - Straight outward paths - Initial heading | | | | | | | | | |
| --- | --- | --- | --- | --- | --- | --- | --- | --- | --- |
| Path | TD vs DS vs WS | | TD vs DS | | TD vs WS | | DS vs WS | |  |
|  | Initial Head. | Ang.  Dev. | Initial Head. | Ang.  Dev. | Initial Head. | Ang.  Dev. | Initial Head. | Ang.  Dev. |  |
| Session 1 | F_(2,62)_ = 12.5014  **p < 0.001** | F_(2,62)_ = 1.893  p = 0.159 | F_(1,45)_ = 6.703  **p = 0.013** | t_(45)_ = 1.188  p = 0.241 | F_(1,44)_ = 2.064  p = 0.239 | t_(44)_ = 1.935  p = 0.059 | F_(1,35)_ = 10.240  **p = 0.003** | t_(35)_ = 0.695  p = 0.492 |  |
| Session 2 | F_(2,62)_ = 5.5611  **p = 0.006** | F_(2,62)_ = 1.818  p = 0.171 | F_(1,45)_ = 2.986  p = 0.091 | t_(45)_ = 1.855  p = 0.070 | F_(1,44)_ = 0.636  p = 0.429 | t_(44)_ = 0.249  p = 0.804 | F_(1,35)_ = 4.558  **p = 0.040** | t_(35)_ = 1.361  p = 0.182 |  |

**Supplementary Material 3.** Homing task - Straight outward paths - Final heading

| 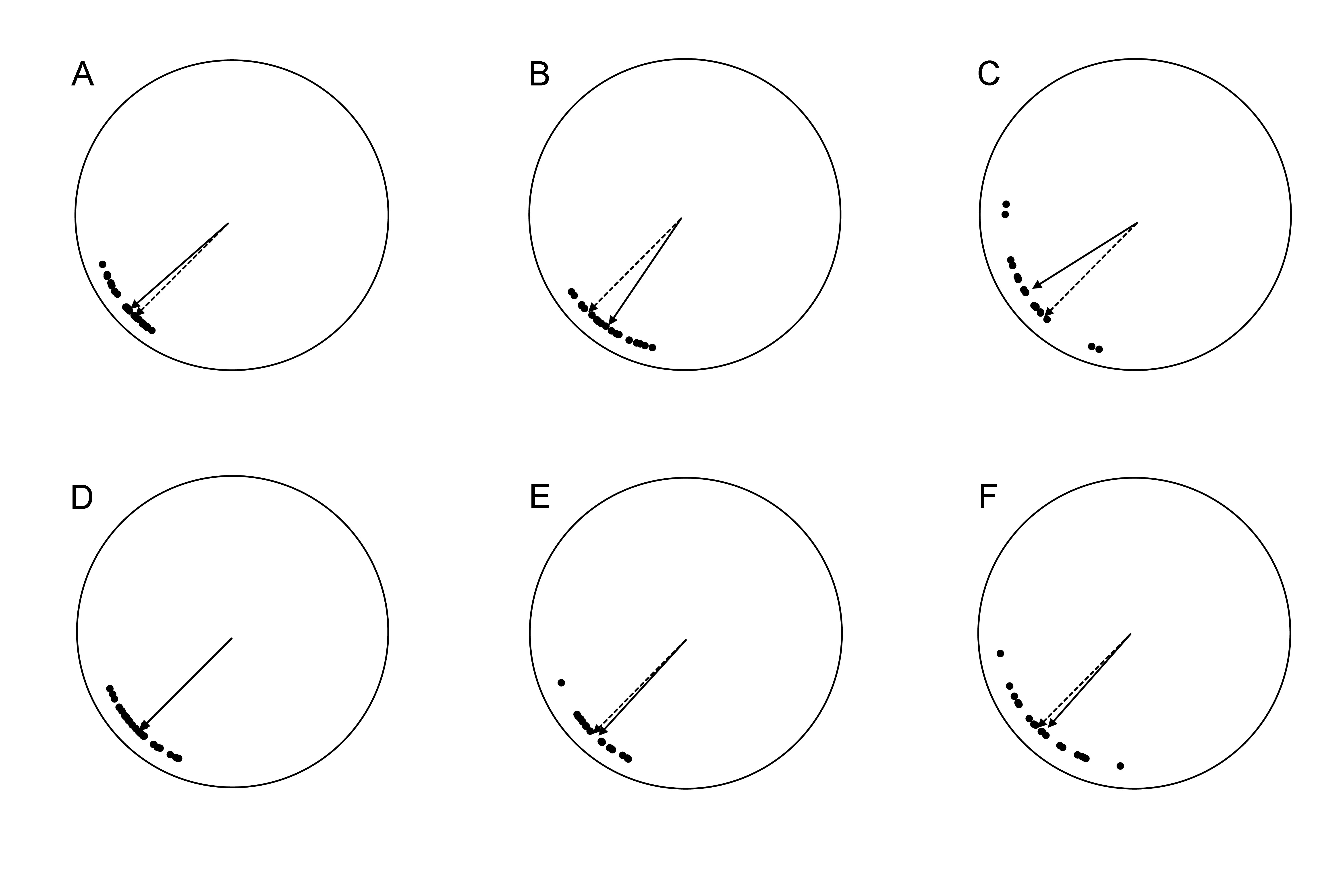 |
| --- |
| **Figure 3.1** Average (solid-line arrow) and individual (dots) final heading direction at the end of the return path, following a straight 7 m outward journey in the homing task. A. TD, Session 1. B. DS, Session 1. C. WS, Session 1. D. TD, Session 2. E. DS, Session 2. F. WS, Session 2. Dashed-line arrow: perfect home direction. |

| **Table 3.1 Within-group results:** Homing task - Straight outward paths - Final heading | | | | | | | | | | | | |
| --- | --- | --- | --- | --- | --- | --- | --- | --- | --- | --- | --- | --- |
|  | TD | | | | DS | | | | WS | | | |
| Path | Final Head. |  | 99%  C.I. | Ang.  Dev. | Final Head. |  | 99%  C.I. | Ang.  Dev. | Final Head. |  | 99%  C.I. | Ang.  Dev. |
| Session 1 | 3.73° | < | 5.17° | 9.87° | **10.48°** | **>** | **8.42°** | 12.73° | 13.09° | < | 14.03° | 19.86° |
| Session 2 | 0.05° | < | 6.09° | 11.57° | 2.44° | < | 7.45° | 11.32° | 3.50° | < | 13.20° | 18.80° |

| **Table 3.2 Between groups comparisons:** Homing task - Straight outward paths - Final heading | | | | | | | | | |
| --- | --- | --- | --- | --- | --- | --- | --- | --- | --- |
| Path | TD vs DS vs WS | | TD vs DS | | TD vs WS | | DS vs WS | |  |
|  | Final Head. | Ang.  Dev. | Final Head. | Ang.  Dev. | Final Head. | Ang.  Dev. | Final Head. | Ang.  Dev. |  |
| Session 1 | F_(2,62)_ = 24.815  **p < 0.001** | F_(2,62)_ = 6.293  **p = 0.003** | F_(1,45)_ = 17.654  **p < 0.001** | t_(45)_ = 2.304  **p = 0.026** | F_(1,44)_ = 4.224  **p = 0.046** | t_(44)_ = 3.423  **p = 0.001** | F_(1,35)_ = 17.593  **p < 0.001** | t_(35)_ = 1.338  p = 0.190 |  |
| Session 2 | F_(2,62)_ = 0.753  p = 0.475 | F_(2,62)_ = 3.551  **p = 0.035** | F_(1,45)_ = 0.510  p = 0.479 | t_(45)_ = 0.460  p = 0.648 | F_(1,44)_ = 0.590  p = 0.447 | t_(44)_ = 2.357  **p = 0.023** | F_(1,35)_ = 0.040  p = 0.842 | t_(35)_ = 1.868  p = 0.070 |  |

**Supplementary Material 4**. Homing task - Angled outward paths - Initial heading

| 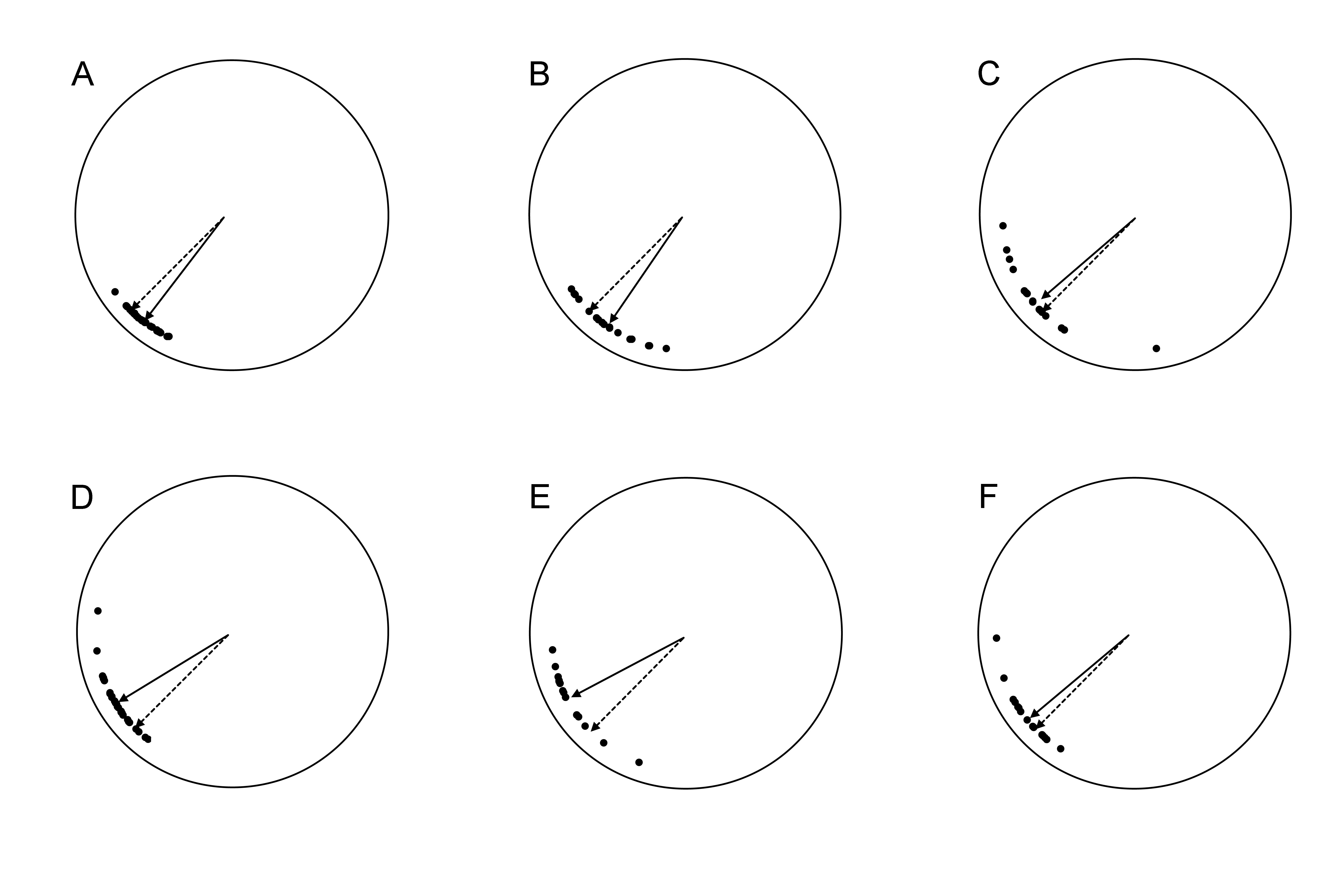 |
| --- |
| **Figure 4.1** Average (solid-line arrow) and individual (dots) walking directions of participants after one meter along the return path, following a two-legged 10 m angled outward journey in the homing task. A. TD, Session 1. B. DS, Session 1. C. WS, Session 1. D. TD, Session 2. E. DS, Session 2. F. WS, Session 2. Dashed-line arrow: perfect home direction. |

| **Table 4.1 Within-group results:** Homing task - Angled outward paths - Initial heading | | | | | | | | | | | | |
| --- | --- | --- | --- | --- | --- | --- | --- | --- | --- | --- | --- | --- |
|  | TD | | | | DS | | | | WS | | | |
| Path | Initial Head. |  | 99%  C.I. | Ang.  Dev. | Initial Head. |  | 99%  C.I. | Ang.  Dev. | Initial Head. |  | 99%  C.I. | Ang.  Dev. |
| Session 1 | **7.52°** | **>** | **3.84°** | 7.37° | **10.12°** | **>** | **9.13°** | 13.76° | 4.49° | < | 14.01° | 19.84° |
| Session 2 | **13.51°** | **>** | **6.79°** | 12.85° | **17.36°** | **>** | **9.68°** | 14.54° | 5.17° | < | 9.17° | 13.40° |

| **Table 4.2 Between groups comparisons:** Homing task - Angled outward paths - Initial heading | | | | | | | | | |
| --- | --- | --- | --- | --- | --- | --- | --- | --- | --- |
| Path | TD vs DS vs WS | | TD vs DS | | TD vs WS | | DS vs WS | |  |
|  | Initial Head. | Ang.  Dev. | Initial Head. | Ang.  Dev. | Initial Head. | Ang.  Dev. | Initial Head. | Ang.  Dev. |  |
| Session 1 | F_(2,62)_ = 11.353  **p < 0.001** | F_(2,62)_ = 2.344  p = 0.104 | F_(1,45)_ = 0.669  p = 0.418 | t_(45)_ = 2.286  **p = 0.027** | F_(1,44)_ = 7.939  **p = 0.007** | t_(44)_ = 1.834  p = 0.073 | F_(1,35)_ = 6.340  **p = 0.016** | t_(35)_ = 0.093  p = 0.926 |  |
| Session 2 | F_(2,62)_ = 7.473  **p = 0.001** | F_(2,62)_ = 4.356  **p = 0.017** | F_(1,45)_ = 0.869  p = 0.356 | t_(45)_ = 1.821  p = 0.075 | F_(1,44)_ = 4.234  **p = 0.046** | t_(44)_ = 1.330  p = 0.190 | F_(1,35)_ = 6.595  **p = 0.015** | t_(35)_ = 3.159  **p = 0.003** |  |

**Description 4.3** When returning to home on the Angled path sessions, participants could also turn “the long way around” requiring an ideal turn of 225° to the left in the Angled path Session 1, or to the right in the Angled path Session 2. In Session 1, only three TD children turned left one time each (TD31, TD49, TD138), and in Session 2, only two TD children turned right one time (TD26, TD191), and one TD child turned right two times (TD167) for a total of only seven long way turns, out of 280 turns. In Session 1, only two participants with DS turned left one time (DS23, DS25), and in Session 2 only two participants with DS turned right one time each (DS19, DS25) for a total of only four long way turns, out of 190 turns. In Session 1, one participant with WS turned left five times (WS17), one participant with WS turned left four times (WS13), and two participants with WS turned left one time (WS5, WS7), and in Session 2, one participant with WS turned right three times (WS2), one participant with WS turned right two times (WS9), and two participants with WS turned right one time (WS7, WS20), for a total of 18 long way turns, out of 180 turns.

**Supplementary Material 5**. Homing task - Angled outward paths - Final heading

| 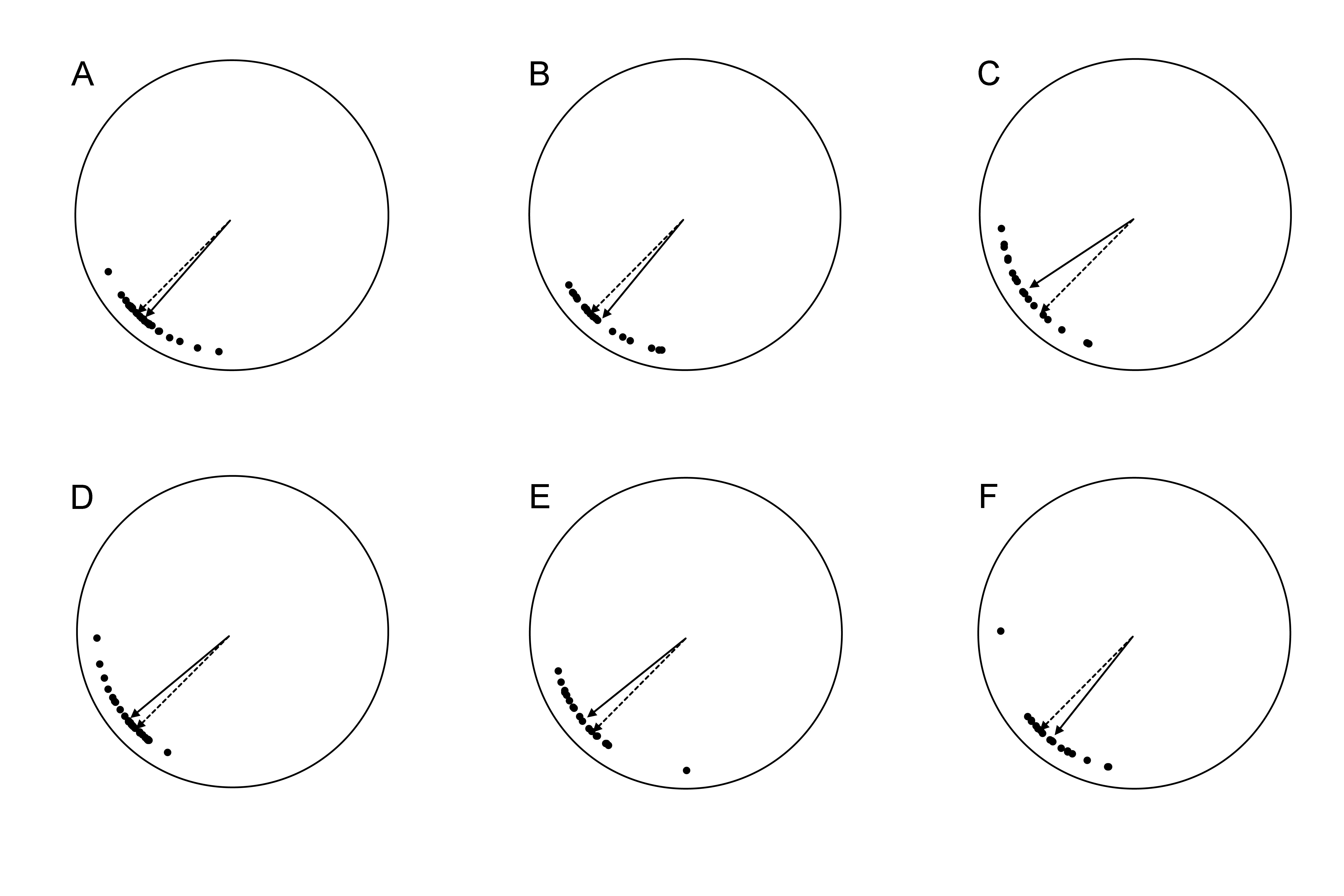 |
| --- |
| **Figure 5.1** Average (solid-line arrow) and individual (dots) final heading direction at the end of the return path, following a two-legged 10 m outward journey in the homing task. A. TD, Session 1. B. DS, Session 1. C. WS, Session 1. D. TD, Session 2. E. DS, Session 2. F. WS, Session 2. Dashed-line arrow: perfect home direction. |

| **Table 5.1 Within-group results:** Homing task - Angled outward paths - Final heading | | | | | | | | | | | | |
| --- | --- | --- | --- | --- | --- | --- | --- | --- | --- | --- | --- | --- |
|  | TD | | | | DS | | | | WS | | | |
| Path | Final Head. |  | 99%  C.I. | Ang.  Dev. | Final Head. |  | 99%  C.I. | Ang.  Dev. | Final Head. |  | 99%  C.I. | Ang.  Dev. |
| Session 1 | 3.66° | < | 6.56° | 12.44° | 5.32° | < | 10.22° | 15.30° | 11.93° | < | 13.12° | 18.70° |
| Session 2 | 5.24° | < | 6.89 | 13.03° | 6.39° | < | 11.09° | 16.52° | 6.35° | < | 12.21° | 17.51° |

| **Table 5.2 Between groups comparisons:** Homing task - Angled outward paths - Final heading | | | | | | | | | |
| --- | --- | --- | --- | --- | --- | --- | --- | --- | --- |
| Path | TD vs DS vs WS | | TD vs DS | | TD vs WS | | DS vs WS | |  |
|  | Final Head. | Ang.  Dev. | Final Head. | Ang.  Dev. | Final Head. | Ang.  Dev. | Final Head. | Ang.  Dev. |  |
| Session 1 | F_(2,62)_ = 14.003  **p < 0.001** | F_(2,62)_ = 5.183  **p = 0.008** | F_(1,45)_ = 0.158  p = 0.693 | t_(45)_ = 0.986  p = 0.330 | F_(1,44)_ = 10.888  **p = 0.002** | t_(44)_ = 3.293  **p = 0.002** | F_(1,35)_ = 8.884  **p = 0.005** | t_(35)_ = 1.943  p = 0.060 |  |
| Session 2 | F_(2,62)_ = 7.520  **p = 0.001** | F_(2,62)_ = 1.021  p = 0.366 | F_(1,45)_ = 0.067  p = 0.797 | t_(45)_ = 1.342  p = 0.186 | F_(1,44)_ = 6.229  **p = 0.016** | t_(44)_ = 1.114  p = 0.271 | F_(1,35)_ = 4.856  **p = 0.034** | t_(35)_ = 0.098  p = 0.922 |  |

**Supplementary Material 6**. Cognitive mapping task - Novel paths - Initial heading

| **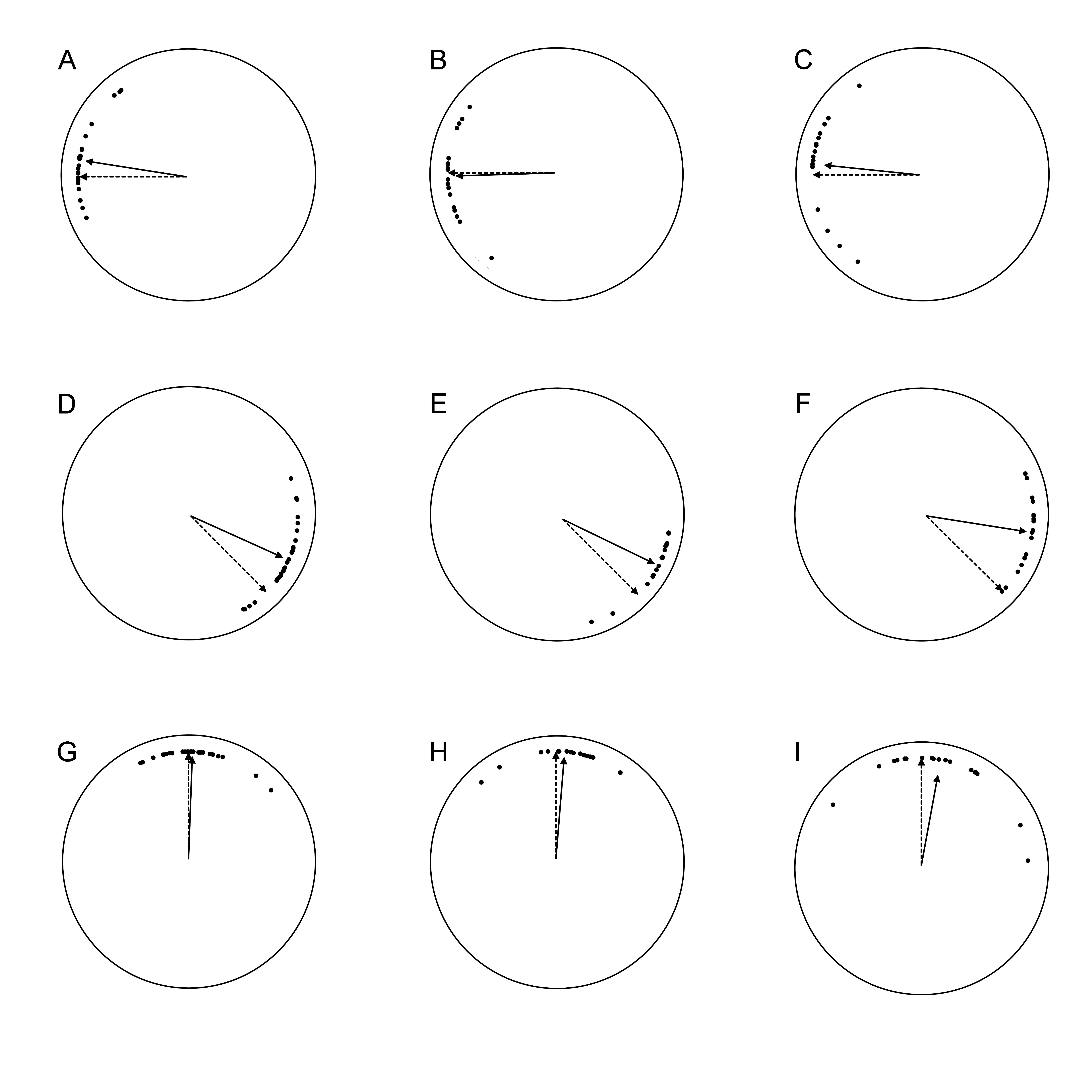** |  |
| --- | --- |
| **Figure 6.1.1** Average (solid-line arrow) and individual (dots) initial heading of participants after walking one meter along the path from the starting object in the cognitive mapping task. A. TD, Bench to Chair. B: DS, Bench to Chair. C: WS, Bench to Chair. D. TD, Chair to Table. E. DS, Chair to Table. F. WS, Chair to Table. G. TD, Table to Shelf. H. DS, Table to Shelf. I. WS, Table to Shelf. | |

| **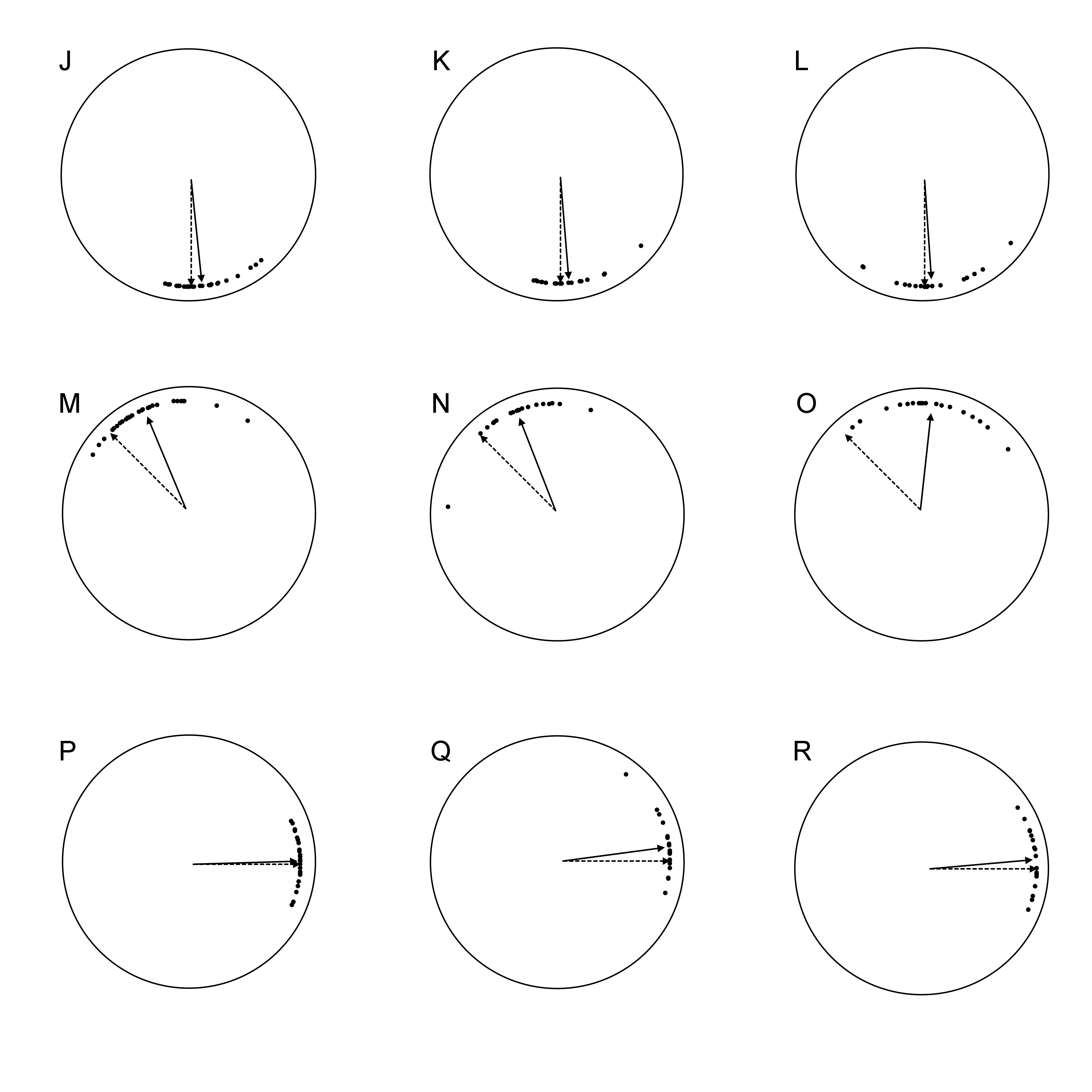** |  |
| --- | --- |
| **Figure 6.1.2** Average (solid-line arrow) and individual (dots) initial heading of participants after walking one meter along the path from the starting object in the cognitive mapping task. J. TD, Shelf to Table. K. DS, Shelf to Table. L. WS, Shelf to Table. M. TD, Table to Chair. N. DS, Table to Chair. O. WS, Table to Chair. P. TD, Chair to Bench. Q. DS, Chair to Bench. R. WS, Chair to Bench. | |

| **Table 6.1 Within-group results:** Cognitive mapping task - Novel paths - Initial heading | | | | | | | | | | | | |
| --- | --- | --- | --- | --- | --- | --- | --- | --- | --- | --- | --- | --- |
|  | TD | | | | DS | | | | WS | | | |
| Path | Initial Head. |  | 99%  C.I. | Ang.  Dev. | Initial Head. |  | 99%  C.I. | Ang.  Dev. | Initial Head. |  | 99%  C.I. | Ang.  Dev. |
| B to C | 8.92° | < | 9.60° | 17.81° | 1.77° | < | 14.87° | 21.53° | 5.90° | < | 18.94° | 25.71° |
| C to T | **20.69°** | **>** | **10.68°** | 19.61° | **19.07°** | **>** | **11.01°** | 16.41° | **35.88°** | **>** | **13.24°** | 18.85° |
| T to S | 2.02° | < | 8.69° | 16.23° | 4.42° | < | 11.66° | 17.30° | 10.49° | < | 23.58° | 30.56° |
| S to T | 5.93° | < | 7.64° | 14.39° | 4.63° | < | 10.44° | 15.60° | 3.57° | < | 15.17° | 21.28° |
| T to C | **21.95°** | **>** | **11.39°** | 20.78° | **23.92°** | **>** | **14.73°** | 21.35° | **51.03°** | **>** | **17.02°** | 23.51° |
| C to B | 1.67° | < | 6.56° | 12.43° | 7.65° | < | 10.38° | 15.53° | 4.89° | < | 10.69° | 15.47° |

| **Table 6.2 Between groups comparisons:** Cognitive mapping task - Novel paths - Initial heading | | | | | | | | | |
| --- | --- | --- | --- | --- | --- | --- | --- | --- | --- |
| Path | TD vs DS vs WS | | TD vs DS | | TD vs WS | | DS vs WS | |  |
|  | Initial Head. | Ang.  Dev. | Initial Head. | Ang.  Dev. | Initial Head. | Ang.  Dev. | Initial Head. | Ang.  Dev. |  |
| B to C | F_(2,62)_ = 2.714  p = 0.074 | F_(2,62)_ = 1.439  p = 0.245 | F_(1,45)_ = 3.223  p = 0.079 | t_(45)_ = 0.670  p = 0.507 | F_(1,44)_ = 0.203  p = 0.654 | t_(44)_ = 1.668  p = 0.102 | F_(1,35)_ = 0.893  p = 0.351 | t_(35)_ = 0.957  p = 0.345 |  |
| C to T | F_(2,62)_ = 9.043  **p < 0.001** | F_(2,62)_ = 3.794  **p = 0.028** | F_(1,45)_ = 0.083  p = 0.775 | t_(45)_ = 0.140  p = 0.890 | F_(1,44)_ = 6.396  **p = 0.015** | t_(44)_ = 2.198  **p = 0.033** | F_(1,35)_ = 7.866  **p = 0.008** | t_(35)_ = 2.424  **p = 0.021** |  |
| T to S | F_(2,62)_ = 1.549  p = 0.221 | F_(2,62)_ = 3.982  **p = 0.024** | F_(1,45)_ = 0.220  p = 0.641 | t_(45)_ = 0.459  p = 0.648 | F_(1,44)_ = 1.355  p = 0.251 | t_(44)_ = 2.531  **p = 0.015** | F_(1,35)_ = 0.511  p = 0.479 | t_(35)_ = 1.885  p = 0.068 |  |
| S to T | F_(2,62)_ = 0.206  p = 0.814 | F_(2,62)_ = 0.794  p = 0.456 | F_(1,45)_ = 0.082  p = 0.776 | t_(45)_ = 0.289  p = 0.774 | F_(1,44)_ = 0.189  p = 0.666 | t_(44)_ = 1.191  p = 0.240 | F_(1,35)_ = 0.028  p = 0.868 | t_(35)_ = 0.836  p = 0.409 |  |
| T to C | F_(2,62)_ = 20.568  **p < 0.001** | F_(2,62)_ = 10.374  **p < 0.001** | F_(1,45)_ = 0.093  p = 0.762 | t_(45)_ = 0.707  p = 0. 483 | F_(1,44)_ = 18.177  **p < 0.001** | t_(44)_ = 4.161  **p < 0.001** | F_(1,35)_ = 12.586  **p = 0.001** | t_(35)_ = 3.377  **p = 0.002** |  |
| C to B | F_(2,62)_ = 1.928  p = 0.154 | F_(2,62)_ = 0.606  p = 0.549 | F_(1,45)_ = 2.030  p = 0.161 | t_(45)_ = 0.643  p = 0.523 | F_(1,44)_ = 0.573  p = 0.453 | t_(44)_ = 1.265  p = 0.212 | F_(1,35)_ = 0.276  p = 0.603 | t_(35)_ = 0.346  p = 0.731 |  |

**Supplementary Material 7**. Cognitive mapping task - Novel paths - Final heading

| **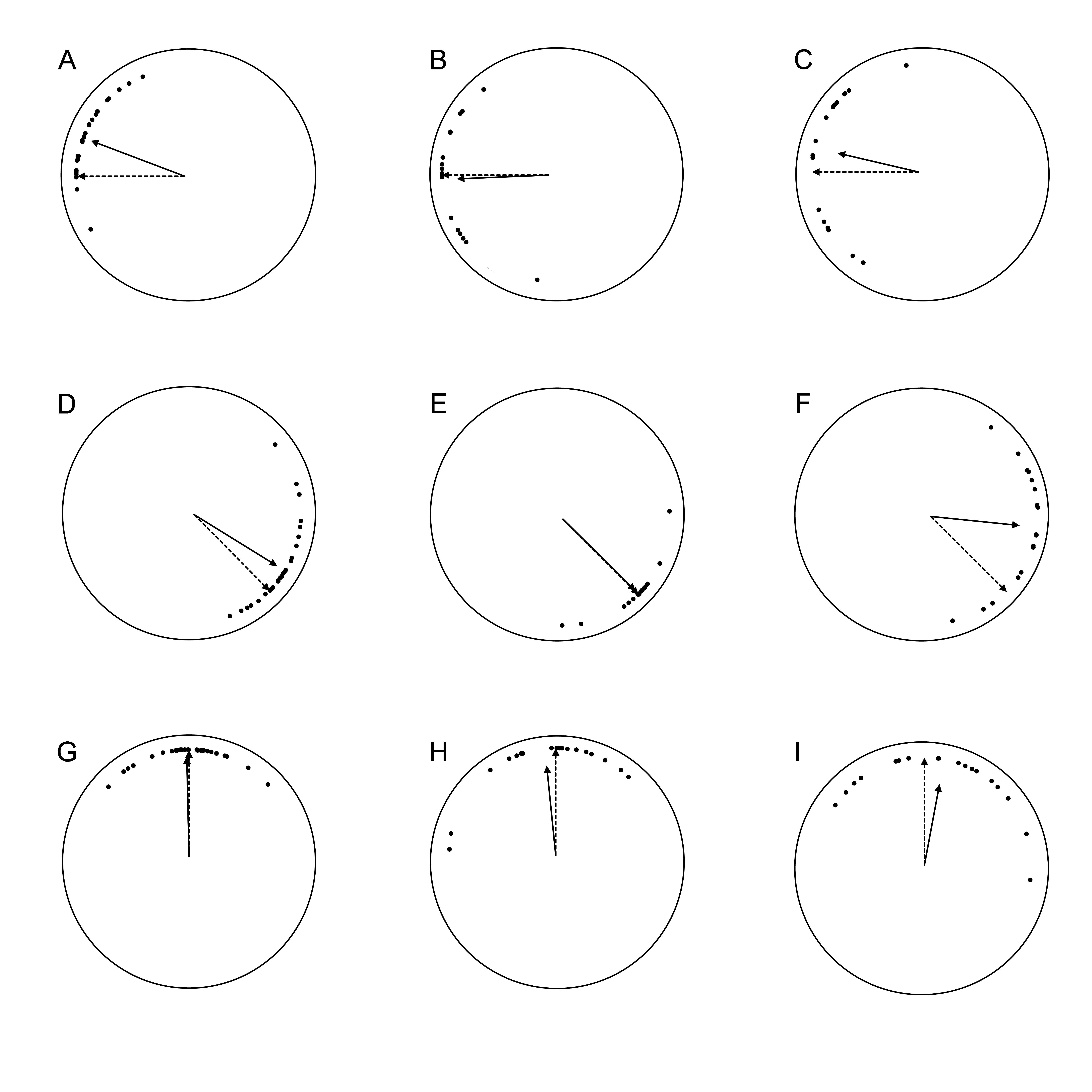** |
| --- |
| **Figure 7.1.1** Average (solid-line arrow) and individual (dots) final heading (at the end of the path) in the cognitive mapping task. A. TD, Bench to Chair. B: DS, Bench to Chair. C: WS, Bench to Chair. D. TD, Chair to Table. E. DS, Chair to Table. F. WS, Chair to Table. G. TD, Table to Shelf. H. DS, Table to Shelf. I. WS, Table to Shelf. |

| **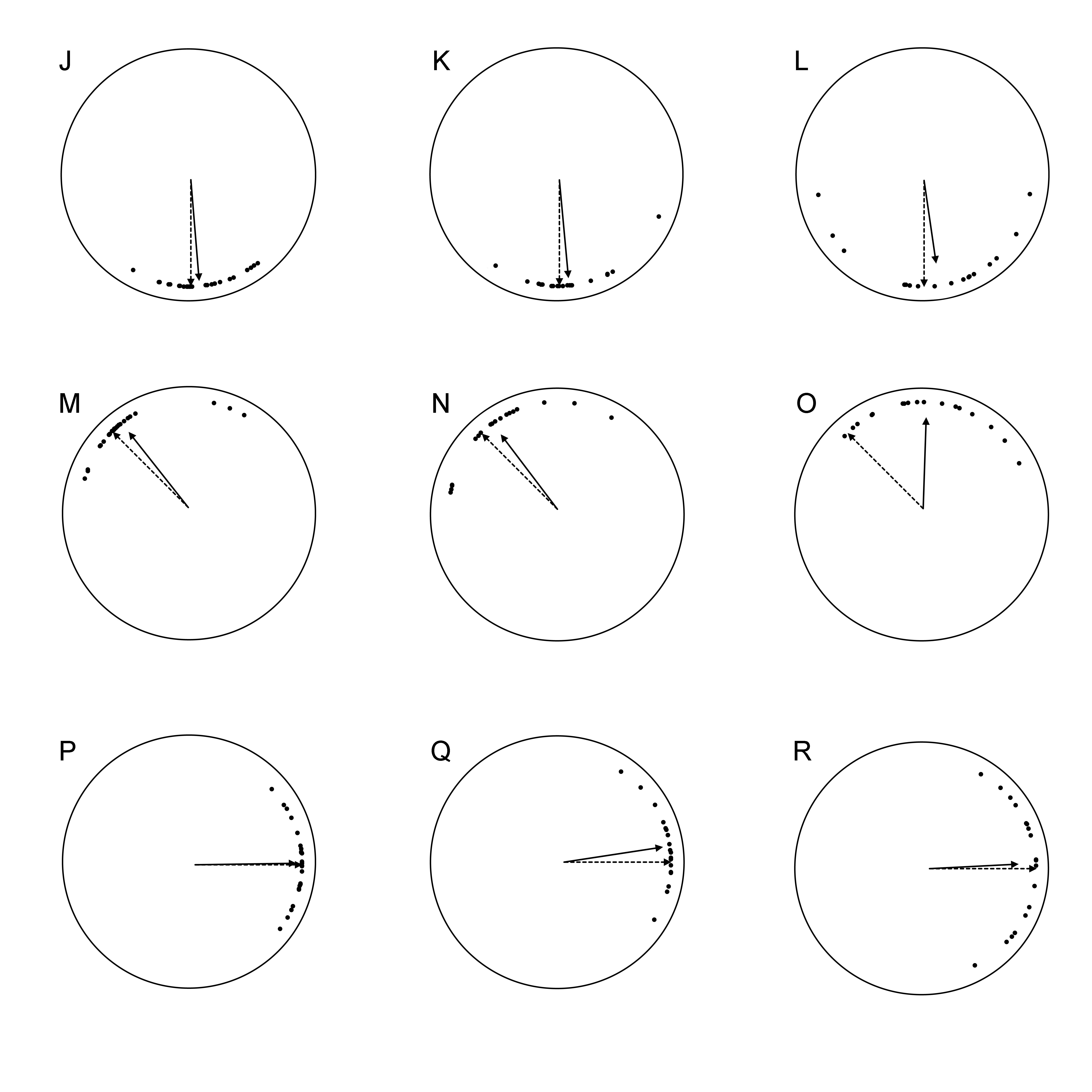** |
| --- |
| **Figure 7.1.2** Average (solid-line arrow) and individual (dots) final heading (at the end of the path) in the cognitive mapping task. J. TD, Shelf to Table. K. DS, Shelf to Table. L. WS, Shelf to Table. M. TD, Table to Chair. N. DS, Table to Chair. O. WS, Table to Chair. P. TD, Chair to Bench. Q. DS, Chair to Bench. R. WS, Chair to Bench. |

| **Table 7.1 Within-group results:** Cognitive mapping task - Novel paths - Final heading | | | | | | | | | | | | |
| --- | --- | --- | --- | --- | --- | --- | --- | --- | --- | --- | --- | --- |
|  | TD | | | | DS | | | | WS | | | |
| Path | Final Head. |  | 99%  C.I. | Ang.  Dev. | Final Head. |  | 99%  C.I. | Ang.  Dev. | Final Head. |  | 99%  C.I. | Ang.  Dev. |
| B to C | **20.57°** | **>** | **11.57°** | 21.08° | 2.58° | < | 22.53° | 30.29° | 13.28° | < | 32.29° | 37.94° |
| C to T | 13.35° | < | 13.41° | 23.97° | 0.29° | < | 12.17° | 17.98° | **38.85°** | **>** | **26.25°** | 33.05° |
| T to S | 1.11° | < | 10.92° | 20.01° | 5.95° | < | 24.09° | 31.84° | 10.40° | < | 33.85° | 39.04° |
| S to T | 4.77° | < | 9.35° | 17.37° | 5.14° | < | 14.53° | 21.09° | 7.77° | < | 31.64° | 37.46° |
| T to C | 6.74° | < | 14.48° | 25.59° | 8.02° | < | 20.24° | 27.87° | **46.73°** | **>** | **23.38°** | 30.36° |
| C to B | 0.80° | < | 10.04° | 18.55° | 8.70° | < | 13.89° | 20.26° | 2.82° | < | 26.56° | 33.32° |

| **Table 7.2 Between groups comparisons:** Cognitive mapping task - Novel paths - Final heading | | | | | | | | | |
| --- | --- | --- | --- | --- | --- | --- | --- | --- | --- |
| Path | TD vs DS vs WS | | TD vs DS | | TD vs WS | | DS vs WS | |  |
|  | Final Head. | Ang.  Dev. | Final Head. | Ang.  Dev. | Final Head. | Ang.  Dev. | Final Head. | Ang.  Dev. |  |
| B to C | F_(2,62)_ = 6.579  **p = 0.003** | F_(2,62)_ = 3.370  **p = 0.041** | F_(1,45)_ = 8.726  **p = 0.005** | t_(45)_ = 0.048  p = 0.962 | F_(1,44)_ = 0.605  p = 0.440 | t_(44)_ = 2.499  **p = 0.016** | F_(1,35)_ = 1.722  p = 0.198 | t_(35)_ = 2.161  **p = 0.038** |  |
| C to T | F_(2,62)_ = 19.865  **p < 0.001** | F_(2,62)_ = 13.237  **p < 0.001** | F_(1,45)_ = 3.849  p = 0.056 | t_(45)_ = 1.655  p = 0.105 | F_(1,44)_ = 8.249  **p = 0.006** | t_(44)_ = 3.618  **p = 0.001** | F_(1,35)_ = 18.099  **p < 0.001** | t_(35)_ = 4.944  **p < 0.001** |  |
| T to S | F_(2,62)_ = 2.448  p = 0.095 | F_(2,62)_ = 5.888  **p = 0.005** | F_(1,45)_ = 0.370  p = 0.546 | t_(45)_ = 1.869  p = 0.068 | F_(1,44)_ = 1.488  p = 0.229 | t_(44)_ = 3.671  **p = 0.001** | F_(1,35)_ = 1.680  p = 0.203 | t_(35)_ = 1.340  p = 0.189 |  |
| S to T | F_(2,62)_ = 0.140  p = 0.870 | F_(2,62)_ = 5.949  **p = 0.004** | F_(1,45)_ = 0.004  p = 0.950 | t_(45)_ = 0.290  p = 0.773 | F_(1,44)_ = 0.117  p = 0.734 | t_(44)_ = 3.188  **p = 0.003** | F_(1,35)_ = 0.062  p = 0.805 | t_(35)_ = 2.348  **p = 0.025** |  |
| T to C | F_(2,62)_ = 23.945  **p < 0.001** | F_(2,62)_ = 8.869  **p < 0.001** | F_(1,45)_ = 0.024  p = 0.878 | t_(45)_ = 1.128  p = 0. 265 | F_(1,44)_ = 21.244  **p < 0.001** | t_(44)_ = 3.889  **p < 0.001** | F_(1,35)_ = 14.907  **p < 0.001** | t_(35)_ = 2.794  **p = 0.008** |  |
| C to B | F_(2,62)_ = 1.120  p = 0.308 | F_(2,62)_ = 5.689  **p = 0.005** | F_(1,45)_ = 1.793  p = 0.187 | t_(45)_ = 0.670  p = 0.506 | F_(1,44)_ = 0.062  p = 0.804 | t_(44)_ = 3.284  **p = 0.002** | F_(1,35)_ = 0.383  p = 0.540 | t_(35)_ = 2.236  **p = 0.032** |  |
